# Liquid-handling Lego robots and experiments for STEM education and research

**DOI:** 10.1371/journal.pbio.2001413

**Published:** 2017-03-21

**Authors:** Lukas C. Gerber, Agnes Calasanz-Kaiser, Luke Hyman, Kateryna Voitiuk, Uday Patil, Ingmar H. Riedel-Kruse

**Affiliations:** 1 Department of Bioengineering, Stanford University, Stanford, California, United States of America; 2 Isaac Newton Graham Middle School, Mountain View, California, United States of America; 3 MYP Dresden International School, Dresden, Germany; 4 University of California Santa Cruz, Santa Cruz, California, United States of America; 5 Georgia Institute of Technology, Atlanta, Georgia, United States of America

## Abstract

Liquid-handling robots have many applications for biotechnology and the life sciences, with increasing impact on everyday life. While playful robotics such as Lego Mindstorms significantly support education initiatives in mechatronics and programming, equivalent connections to the life sciences do not currently exist. To close this gap, we developed Lego-based pipetting robots that reliably handle liquid volumes from 1 ml down to the sub-μl range and that operate on standard laboratory plasticware, such as cuvettes and multiwell plates. These robots can support a range of science and chemistry experiments for education and even research. Using standard, low-cost household consumables, programming pipetting routines, and modifying robot designs, we enabled a rich activity space. We successfully tested these activities in afterschool settings with elementary, middle, and high school students. The simplest robot can be directly built from the widely used Lego Education EV3 core set alone, and this publication includes building and experiment instructions to set the stage for dissemination and further development in education and research.

Robotics and automation significantly advance the life sciences, e.g., via academic, industrial, and pharmaceutical liquid-handling robots [1,2] and open source approaches [3]. Consequently, formal and informal education must convey these concepts. The Next Generation Science Standards (NGSS) and other national initiatives promote cross-disciplinary approaches for science, technology, engineering, and math (STEM) learning [4,5]. Many engaging and successful educational approaches to robotics exist, such as Lego Mindstorms or the FIRST Robotics Competition [6–11]. Naturally, these activities foremost focus on mechanical engineering, computer programming, and soft skills like teamwork. To a lesser extent, they are used to support experiments in Natural Science and Math education [12]. Crucially, integration of equivalent robotics approaches with the life sciences and chemistry for K–12 and college education are lacking, hence we expect significant value in bridging this gap.

Here, we designed simple yet powerful liquid-handling modules that can be integrated into various Lego Mindstorms robots (Fig 1), enabling a variety of engaging and educational life science experiments (Fig 2) (for building and experiment instructions see S1 Text, S1–S3 Movies). The most basic pipetting robot (Fig 1A) is built solely from parts in the standard Educational EV3 Lego kit (45544) (one robot per kit) and less than US$5 in plasticware, enabling easy reproduction. Up to 20 cuvettes are reversibly fixed with double-sided tape onto a motorized 1-D trolley (Fig 1A and 1B). Liquids are delivered and removed via a standard 1-ml plastic syringe with a pipette tip (Fig 1C), which is Lego compatible with minor modifications (Fig 1D). A motorized crankshaft drives the syringe plunger (Fig 1E), which can hold and deliver approximately 720 μl of liquid at a time. More complex calibration and delivery procedures achieve volumes down to 7 μl (20% precision, 30% accuracy, S1_2.2 Text). This liquid-handling module is inspired by professional pipettors (Fig 1E inset). This module can be operated by hand (Fig 3A) or incorporated into the robot (Fig 1A), in which another crankshaft motor lifts and lowers this module relative to the cuvettes. More advanced robot and pipette designs (Fig 1F, S1_3 Text) [13] enable even better liquid handling and 2-D operation with multiple 6-, 24-, or 96-well plates standard plasticware. Here, the syringe is driven by a linear rail system (Fig 1G). This enables convenient delivery of droplets of various sizes down to 2.5 μl (25% precision, 8% accuracy) using a 1-ml syringe and down to 0.15 μl (15% precision, 8% accuracy) using a 25-μl syringe, which is better than what we could obtain with a professional P2 pipette (Fig 1H and 1I, S1_3.3.3 Text). In general, the quality of liquid handling depends on many factors, including fluid characteristics, piston diameter, size of the outlet hole, tip coating, and the impulse of piston advancement [14]. Using sensors for homing enables positioning of the pipette tip with spatial precision of ±2 mm. These robots went through multiple design iterations, are mechanically stable over at least 1,000 pipetting cycles, and are controllable in real time by pressing buttons (Fig 1A) or by preprogrammed routines.

**Fig 1:**
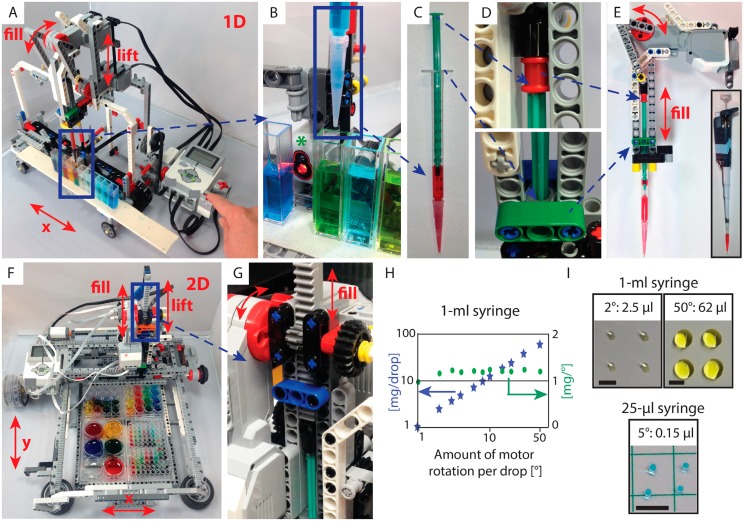
Liquid-handling Lego robots enable hands-on learning of modern biotechnology concepts. (**A**) The 1-D robot constructed from the educational EV3 kit can handle up to 20 standard cuvettes (**B**). A standard 1-ml syringe (**C**) is easily modified for Lego compatibility (**D**). The motorized crankshaft pipette head (**E**) is inspired by professional laboratory pipettes (inset). (**F**) An advanced 2-D robot can handle up to four 96-well plates, in which a linear rail system (**G**) enables precise droplet delivery (**H**). (**I**) Drop volumes for 1-ml and 25-μl syringes using the linear rail system (G) are calibrated from images against drops obtained with standard pipettes (Inset E); scale bars: 5 mm.

**Fig 2:**
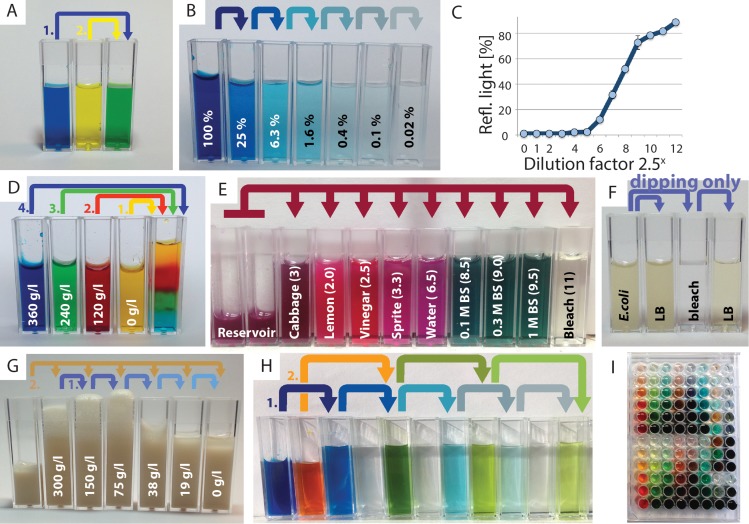
Examples of science experiments and activities that are enabled by the Lego liquid-handling robot. (**A**) Transfer and mixing of colored water. (**B**) Serial dilution of colored water. (**C**) Intensity readouts from a dilution series similar to (B) via the Lego color sensor (*in Fig 1B) (*n* = 6 measurements at each point, error bars are 1 standard deviation [stdev]). (**D**) Liquids of different salt densities do not mix if gently layered in order. (**E**) Red cabbage juice as a pH indicator of various household liquids (pH in brackets). BS, baking soda. (**F**) Sterilization of the syringe prevents bacterial growth and avoids the need for disposable tips. LB, lysogeny broth. (**G**) Identification of the optimal sucrose concentration for yeast growth. (**H,I**) Automated loops and complex routines programmed with the Lego software for complex mixing protocols in cuvettes and multiwell plates.

**Fig 3:**
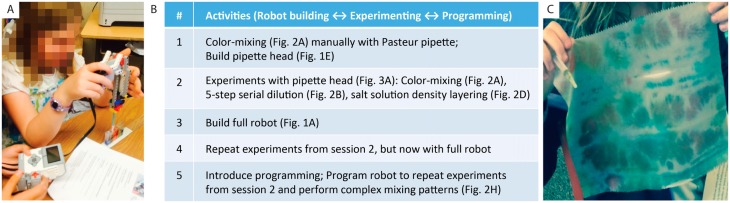
User studies in afterschool settings combine robot building, science experiments, and programming. (**A**) Children 10–13 years old built and explored the functionality of these robots by performing experiments. (**B**) Activity progression over five sessions, each lasting about 90 minutes. (**C**) Example of self-initiated student activity using classic Pasteur pipettes and robot pipettor to make colored patterns on paper.

We developed a set of basic experiments and activities for the 1-D robot that cover a wide variety of science experiments and topics with standard, low-cost plasticware and common household or school consumables, ensuring accessibility and safe use (Fig 2). Mixing of colored liquids from two reservoirs into a third reservoir demonstrates basic liquid handling (Fig 2A). Serial dilutions illustrate the concept of concentration (Fig 2B). Aligning the cuvette with the Lego color sensor on the robot (Fig 1B) showcases the functionality of spectrophotometers (Fig 2C). Colored liquids with distinct salt densities can be sequentially layered in a single cuvette to model buoyancy (Fig 2D). Cabbage juice constitutes a pH indicator for various liquids, e.g., water, lemon juice, and bleach (Fig 2E). Sterilization is achieved by uptake and release of 80% alcohol or 10% bleach multiple times; in contrast, cross-contamination occurs without sterilization (Fig 2F). Adding baker’s yeast to serially diluted sugar solutions leads to foam formation due to CO_2_ production and demonstrates optimal growth conditions at intermediate nutrient concentration (Fig 2G). Automated dilution series enable complex mixing and dilution series in cuvettes (Fig 2H) or multiwell plates (Fig 2I). Similar experiments are currently used in various school settings using Pasteur pipettes by hand.

In order to assess the potential for education and wider dissemination, we carried out two user studies (Fig 3A, S1_6 Text) focusing on the 1-D robot (Fig 1A). Specifically, we investigated whether these activities could successfully integrate robot building, programming, and wet-science experiments in an engaging way, whether elementary and middle school students could successfully complete these activities, and what the required time frame would be. Among the available science activities (Fig 2), we focused on liquid mixing, dilution series, and liquid density (Fig 2A, 2B and 2D), which aligns well with middle school learning content [5]. The robotic activities focused on building and programming, as is common in afterschool settings [11]. Given the unknowns associated with a first deployment, we significantly guided all activities using worksheets. We designed a curriculum (Fig 3B) that progresses from classic experiments with Pasteur pipettes, to real-time control of the pipettor module held by hand while pushing buttons (Fig 3A), to real-time control of the robot while pushing buttons (Fig 1A), all the way to programming the robot. Partial repetition of science experiments between sessions intended to deepen the concepts and to emphasize the differences between humans and liquid-handling robots, e.g., higher precision (session 2 versus session 4) and automation of repetitive tasks (session 2 versus session 5). Assessment was based on observed student activities as well as evaluation of worksheets, posttests, questionnaires, and self-reported learning.

Eight elementary school students (seven used for the study according to institutional review board (IRB) guidelines; 10–11 years old; all Girl Scouts, 4/7 had previous Lego Mindstorms experience) worked with the robots in groups of two over five 90-minute afterschool sessions (Fig 3B, S1_6.2 and S1_7 Text). In session 1, two instructors demonstrated a fully automated dilution series (Fig 2B) and explained the general importance of liquid handling and robotics for the life sciences. The participants manually handled and mixed water with food coloring (Fig 2A) using a plastic Pasteur pipette, then built their own robot pipette module (Fig 1E). In session 2, the participants used their module to carry out the color-mixing (Fig 2A), five-step serial dilution (Fig 2B), and salt solution density layering (Fig 2D) experiments. One partner always held and aligned the pipette with the cuvettes, while the other operated the two push buttons on the Lego brick to manipulate the liquids (Fig 3A). During session 3, each group built one or two of the five main structural robot modules (Fig 1A, S1_2.1 Text); the instructors helped assemble all modules to demonstrate one complete robot. For session 4, each group was provided with a completed robot. Groups then repeated all experiments from session 2 by manually controlling all motors via the buttons on the Lego brick (Fig 1A). During session 5, the participants programmed the robot to perform simple and complex dilution series, for example, through programs with inner and outer loop skipping of every second or third cuvette to generate patterns (Fig 2H). Here, participants also had to measure and convert degrees of motor rotation into linear distance between cuvettes. The course concluded with a postactivity quiz.

Participant feedback and our observations attest to the utility of these activities (S1_6.2 Text). Two instructors for eight participants working in pairs were able to provide sufficient guidance, to correct mistakes, and to discuss content in more depth. Participants were motivated and enjoyed these activities, e.g., they frequently repeated experiments with other (favorite) colors instead of moving directly on to the next experiment, they integrated sounds into their code on their own initiative, and multiple participants used the Pasteur and the Robot pipette to make colorful patterns on tissues that had been provided to clean up spills (Fig 3C). After the course, the participants rated the difficulty of each activity on a 1–5 Likert scale (very easy, easy, medium, hard, very hard): programming (2.8 ± 1.5) (always mean ± standard deviation [stdev]), building the robots (2.5 ± 0.8), density layering (2.5 ± 1.4), dilution series (1.8 ± 0.4), and color mixing (1.3 ± 0.5). Completed worksheets and quizzes demonstrated reasonable associations about why scientists pipette fluids (“liquids don’t need to be touched”), what robots are good for (“more controlled”), and the disadvantages of robots (“can have glitches”). The overall rating of the course on a scale of 1–5 (very bad–brilliant) was 4.2 ± 1.0. When asked what they had learned, all participants pointed to robots and programming; when asked what they liked, they pointed to both robots and the liquid experiments. We also tested whether density layering experiments would increase conceptual understanding of why objects float or sink, and of the three participants who had answered the prequestion incorrectly, two gave the correct answer afterwards.

A second user study included nine middle school students (aged 11–13 years; both genders, 8/9 had previous experience with Lego Mindstorms, S1_6.3 Text) working in groups of two or three over 16 afterschool sessions (~30 hours total). This course was taught by a single middle school teacher; it followed a similar layout as before (Fig 3B), but participants worked under less supervision, all groups built their own robot, participants had more time for self-motivated side projects including individual changes to the robot design, and the teacher inserted several lectures about liquids, densities, and dilution factors (S1_6.3 Text).

Outcomes were generally consistent with the first study. This course was rated 4.2 ± 0.4 on a scale of 1–5 (strongly unfavorable to strongly favorable), and all activities were reported as having medium difficulty (2.1 ± 0.9 to 3.3 ± 0.7) on a scale of 1–5 (very easy–very hard). All students self-reported that they learned something new—7/9 mentioned wet-work (“I learned about salt density” or “I learned about serial dilution”). When asked specifically what they learned about liquid solutions, 5/9 pointed to the density concept (“liquids with higher salinity fall”), 2/9 to solutions in general (“a solution requires care to make”), and the remaining 2/9 answered “nothing” and “It's not easy.” When asked posttest what would happen if two liquid samples of different color and salt content were put over each other in either sequence, 9/9 provided the correct outcome, and 6/9 correctly referred to the different densities. Students also self-reported to have gained competency in building (7/9) and programming (8/9). These activities seemed to have broadened students’ perception of what robots can do, extending from classic engineering into science (“I learned that robotics…can be used in different ways”; “it is a lot more impressive than moving robots.”) When asked what they liked most, all activities were mentioned (e.g., “I liked programming our first experiment”; “I like the density layers sessions the best”; “I liked building and programming the robot”), suggesting that robotics and wet-experimentation can be bridged successfully.

The impact of these activities is extendable in various ways in the future. Students could have more freedom, e.g., develop their own robot and pipette designs. Over a summer, three high school students implemented different robots, including one with moving plates and a stationary pipettor (S1_5 and S1_6.1 Text). Here, we identified as a major design challenge to build a pipettor module that is high performance in liquid handling but is also small enough to be supported by a multiaxis gantry robot. Students could perform quantitative experiments, utilizing more complex control and data-logging capabilities. For example, we used the Lego light sensors to measure the concentrations in a dilution series (Figs 1B, 2B and 2C), but where the limitations of this sensor required careful alignment to obtain reproducible readings (S1_2.4.6 Text). The compatibility with standard plasticware (Fig 2A and 2I) and our washing and sterilization techniques (Fig 2F) might even be sufficient for certain research applications and citizen science [15]. Whether a Lego Mindstorms robot could pick up and release disposable pipette tips with sufficient reliability is doubtful but an open question. Extensions beyond Lego could target lower cost or higher precision; furthermore, remotely controlled labs could be supported [13,16].

In summary, our Lego-based liquid-handling robots combine with a versatile set of science experiments to safely and robustly meet important cross-disciplinary endpoints, integrating robotics, biology, chemistry, and hands-on learning. Our initial user studies point to the validity of our approach; future studies should focus on larger cohort sizes, including control groups, more teachers, and dissemination and utility beyond afterschool programs. The foundation for these robots, the EV3 kit (~US$380), is already available in many schools, while additional reagents are low cost (~US$5) and easily accessible. A minimalistic activity focusing on the hand-held robot pipette (Fig 1A) and simple mixing and density experiments (Fig 3B; sessions 1 and 2) requires even less Lego parts, aligns with the NGSS sixth and eighth grade [5], and could be done within 2–3 hours. These activities may also help to extend Seymour Papert’s Mindstorms vison [6] to the life sciences and chemistry. We invite other stakeholders such as teachers, students, DIY learners, and educational and life science researchers to use, disseminate, and further develop these robots via open-source instructions and protocols.

## Materials and methods

### Lego

The 1-D robot only requires parts included in the Lego Mindstorms EV3 Education edition (Lego Mindstorms EV3 Core Set 45544, Amazon B00DEA55Z8; US$380). Note that the EV3 Home edition of this kit would require additional pieces to build this robot.

### Software

The Lego Mindstorms EV3 Home Edition software (free download on Lego website: http://www.lego.com/en-us/mindstorms/downloads/download-software) was used to program the robots to run experiments. The software is based on LabView, a widely used commercial software. To upload code to the robot, a PC (Mac or Windows), tablet (iOS or Android), or smartphone (iOS or Android) is required. Building instructions were made with the free Lego software, Lego Digital Designer (http://ldd.lego.com). Both programs are available for PC and Mac. The control software is also available for iOS and Android.

### Non-Lego parts

Our design included cuvettes (Standard Cuvette Polystyrene Macro 3.5 ml, Amazon B00T5A64PQ), syringes (Plastic Syringe, Luer Slip, 1 ml, Amazon B00BQLJFYE), tips (Dispensing Needle, Plastic Tapered Pink 20 ga 0.024id x 1.25", Amazon B001QQ9QH0), 6-, 24-, and 96-well plates (Amazon B0177QVE1S, B0177QVILY, and B0177QVE7C, respectively), food color (AmeriColor Beginner Soft Gel Paste Food Color 4 Pack Kit, Amazon B002L3RV9C), a ruler (for mechanical support; School Smart Plastic Ruler, Amazon B003V1HDSM), double-sided carpet tape (XFasten Indoor Carpet Tape Double sided, Amazon B0141L81GS), and instant glue (Gorilla Super Glue Gel, Amazon B00CJ5EO2E). To install the syringe into the pipetting head, two simple modifications were made: some plastic was cut from the top of the syringe holder, and the top of the plunger was removed and glued to a red Lego peg included in the kit (Technic 32054 [pin 3L with friction ridges lengthwise and a stop bush]). Cuvettes were mounted on the 1-D robot via double-sided tape. For smallest droplet volumes, a 25-μl Hamilton glass pipette with steel plunger was used.

### Consumables

Readily available reagents included salt, sugar, and baker’s yeast. Salt solutions for the density-layering experiments were prepared by dissolving 18.0 g, 12.0 g, and 5.9 g sodium chloride in 50 ml water 100%, 67%, and 33% solutions, respectively). For the pH experiments, 300 g of red cabbage were blended with 300 ml of tap water in a Bullet blender for 30 seconds. The mixture was boiled for 10 minutes on medium heat. After cooling, the mix was filtered through a round coffee filter, yielding ~300 ml of a deep purple solution. As control, the pH of analyte solutions was also measured with pH indicator strips (Fisherbrand Plastic pH Strips).

### User studies and IRB approval

The 1-D robot was tested in two independent user studies. The first test group consisted of eight fifth-grade girls. Five 90-minute-long afterschool sessions were conducted over two months. The second test group consisted of nine middle-school students who built, programmed, and used the 1-D robot in 16 two-hour sessions spread over six weeks. In both user studies, we evaluated the utility of the robot and lesson plans with subjective observations, worksheets, and final questionnaires. All human subject studies were performed in accordance with Stanford IRB-18344. All parents gave consent. One child in the first study did not provide assent. This child participated in all activities but her data were excluded from analysis.

### Ethics statement

All human subject studies were performed in accordance with Stanford IRB-18344. None of the authors has any relationship with The Lego Group that would constitute a conflict of interest.

## Supporting information

S1 Text Supplementary text. Overview supplements; Building instructions; Experiment instructions; User studies; Work sheets.
(PDF)

S1 Data CAD files for building lego robots. (ZIP)

S2 Data Software for running lego robots. (ZIP)

S1 Movie Overview movie. (MP4)

S2 Movie 1D_robot.mp4 Movie demonstrating the 1D robot.
(MP4)

S3 Movie 2D_Robot.mp4 Movie demonstrating the 2D robot.
(MP4)

## Abbreviations

IRB: institutional review board
NGSS: Next Generation Science Standards
stdev: standard deviation
STEM: science, technology, engineering, and math
