# Supplementary material for: Liquid-handling Lego robots and experiments for STEM education and research
Source: PLoS Biol. 2017 Mar 21;15(3):e2001413. doi: 10.1371/journal.pbio.2001413 (PMC5360201; doi:10.1371/journal.pbio.2001413)
Supplement: S1 Text — Overview supplements; Building instructions; Experiment instructions; User studies; Work sheets. (PDF) [file pbio.2001413.s001.pdf]

SUPPLEMENTARY MATERIAL

for

Liquid-handling Lego robots for STEM education

Lukas C. Gerber, Agnes Calasanz-Kaiser, Luke Hyman, Kate Voitiuk, Uday Patil, Ingmar H. Riedel-Kruse

Table of Contents

1. OVERVIEW 3

1.1. LIST OF SEPARATE FILES 3
MOVIES: 3
CAD FILES: 3
CODE FILES: 3

2. 1D ROBOT - BUILDING PLAN, INSTRUCTIONS, AND EXPERIMENTS 5

2.1. BUILDING 1D ROBOT USING CAD FILE AND CLOSE-UP PHOTOS 5
2.1.1. 1_PIPETTE MODULE 7
2.1.2. 2_BACK MODULE 8
2.1.3. 3_FRONT MODULE 8
2.1.4. 4_TOP MODULE 9
2.1.5. 5_TROLLEY MODULE 9
2.1.6. 6_BRICK MODULE AND 7_SENSOR MODULE 9
2.1.7. 8_GEARS MODULE 10
2.2. PERFORMANCE OF LIQUID DISPENSING AND ROBOT STABILITY 10
2.3. SOFTWARE FILES 1D ROBOT 11
2.4. EXPERIMENTS 1D ROBOT 12
2.4.1. FIXING THE CUVETTES (EASY, 5 MIN) 12
2.4.2. MANUAL CONTROL OF THE ROBOT (EASY, 5 MIN) 12
2.4.3. PROGRAMMING OF THE ROBOT (EASY, 30 MIN) 12
2.4.4. MIXING EXPERIMENT (EASY, 5 MIN) 12
2.4.5. DILUTION SERIES EXPERIMENT (EASY, 10 MIN) 13
2.4.6. COLOR READOUT (MEDIUM, 30-60 MIN) 13
2.4.7. DENSITY LAYER EXPERIMENT (MEDIUM, 30 MIN) 14
2.4.8. PH INDICATOR EXPERIMENT (MEDIUM, EXTENDED PREPARATION, 30 MIN) 14
2.4.9. YEAST GROWTH (ADVANCED, 60 MIN) 14
2.4.10. DISINFECTION FROM E.COLI (ADVANCED, SPECIAL EQUIPMENT REQUIRED, 30 MIN) 15
2.4.11. SKIPPING CUVETTES (EASY, 10 MIN) 15
2.4.12. SMALLER DROPS (ADVANCED, 60 MIN) 16

3. 2D ROBOT - BUILDING PLAN, INSTRUCTIONS, AND EXPERIMENTS 17

3.1. BUILDING 2D ROBOT USING CAD FILE AND CLOSE-UP PHOTOS **17**
3.1.1. SYRINGE 18
3.1.2. LIFT 18
3.1.3. PREPARE THE SYRINGE WITH BLUE "LIGHT SENSOR FLAG" 19
3.1.4. ADD THE SYRINGE 19
3.1.5. MERGE SYRINGE AND LIFT PARTS INCLUDING THE SYRINGE 21
3.1.6. CAP 21
3.1.7. FRAME 22
3.1.8. CART 22
3.2. SOFTWARE FILES 2D ROBOT **23**
3.3. EXPERIMENTS 2D ROBOT **23**
3.3.1. ANALOG TO 1D 23
3.3.2. STANDARD LAB WARE: 6-, 24-, AND 96 WELL-PLATES 23
3.3.3. MODES AND PERFORMANCE OF LIQUID DELIVERY 24

4. EXAMPLE LESSON PLAN (AS DEPLOYED IN ELEMENTARY SCHOOL) 27

4.1. LESSONS PLAN **27**
4.2. EXAMPLE WORKSHEETS **27**

5. OTHER ROBOT DESIGNS 28

6. DESCRIPTION AND OUTCOMES OF THE USER STUDIES 30

6.1. FIRST USE CASE: HIGH SCHOOL STUDENTS SUMMER RESEARCH PROJECT **30**
6.2. SECOND USE CASE: ELEMENTARY SCHOOL 5TH GRADE GIRL SCOUTS **31**
6.3. THIRD USE CASE: MIDDLE SCHOOL STUDENTS **35**
6.4. CONCLUSIONS ALL USER TESTING **40**

7. EXAMPLE WORKSHEETS AS USED WITH GIRL SCOUTS (ELEMENTARY SCHOOL) 41

DAY1: OVERVIEW, MANUAL MIXING, BUILDING OF PIPETTING HEAD MODULE **41**
DAY 2: USE OF ROBOT'S PIPETTE HEAD MODULE ONLY **42**
DAY 3: BUILDING COMPLETE ROBOT **45**
DAY 4: MANUAL CONTROL OF FULL ROBOT **46**
DAY 5: PROGRAMMING **54**

1. Overview

The supporting materials to this paper contain detailed information about building the robots, suggested experiments, material and methods, proposed worksheets, and user studies, and provide illustrative movies. This file also refers to all other files such as movies, code, and CAD.

1.1. List of separate files

Movies:

- ***Overview.mp4***
  Summary of the 1D and 2D robots including some experiments (1D: setup, actuation of motors, manual control, programmed loops, color mixing, dilution series, density layers. 2D: Well-plates, dilution series, small drops).
- ***1D_robot.mp4***
  Color mixing, dilution series, and density layers in real time. Showing all Lego parts and additional materials required for the experiments and briefly introducing the Lego programming software.
- ***2D_robot.mp4***
  Shows the 2D robot in action performing a reset of all motors and cleaning of the syringe. This is followed by a dilution series in 24 well-plates and a density layer into a cuvette are shown. Also the two ejection modes (jet vs. dip-in) are demonstrated.

CAD files:

- *1D_0_all_in_one.lxf*
- *1D_1_Pipette.lxf*
- *1D_2_Back.lxf*
- *1D_3_Front.lxf*
- *1D_4_Top.lxf*
- *1D_5_Trolley.lxf*
- *1D_6_Brick.lxf*
- *1D_7_Sensor.lxf*
- *1D_8_Gears_(with_2_Back).lxf*
- *1D_Part_list.xlsx*
- *2D_1_Syringe.lxf*
- *2D_2_Lift.lxf*
- *2D_3_Cap.lxf*
- *2D_4_Frame.lxf*
- *2D_5_Cart.lxf*
- *2D_Part_list.xlsx*

Code files:

- *1D.ev3*
  Code to be run with the Lego Mindstorms software for the 1D robot.

- *2D.ev3*
  Code to be run with the Lego Mindstorms software for the 2D robot.

2. 1D robot - Building plan, instructions, and experiments

The 1D robot presented here can be built from a single Lego Mindstorms kit (EV3 Core Set 45544, Amazon #B00DEA55Z8; US $380) and some easy accessible additional parts (<US $5). This robot can pipette liquids and address 20 cuvettes placed on a ruler. The three motors included in the Lego set can be controlled over the free Lego software. The following photos and separate files will allow other users to replicate and use the 1D robot presented here.

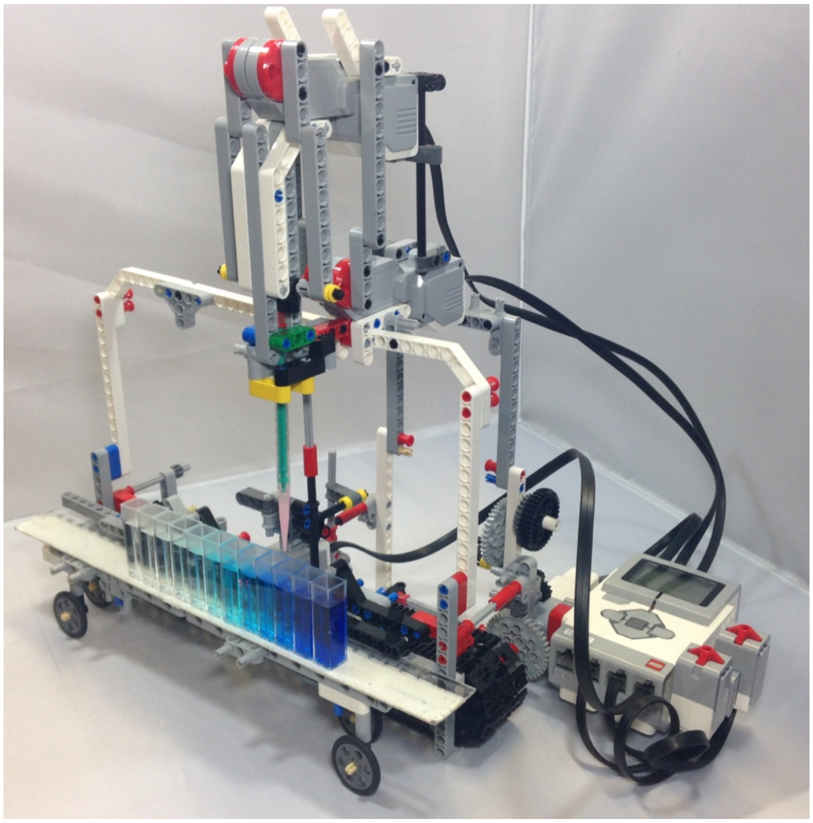

Fig S1. 1D robot with completed dilution series.

Additional material suggested for the experiments below: Cuvettes (Standard Cuvette Polystyrene Macro 3.5 mL, Amazon #B00T5A64PQ), syringes (Plastic Syringe, Luer Slip, 1 mL, Amazon #B00BQLJFYE), tips (Dispensing Needle, Plastic Tapered Pink 20 ga 0.024id x 1.25", Amazon #B001QQ9QH0), food color (AmeriColor Beginner Soft Gel Paste Food Color 4 Pack Kit, Amazon #B002L3RV9C), a ruler (for mechanical support; School Smart Plastic Ruler, Amazon #B003V1HDSM), double-sided carpet tape (XFasten Indoor Carpet Tape Double sided, Amazon #B0141L81GS), and instant glue (Gorilla Super Glue Gel, Amazon #B00CJ5EO2E).

2.1. Building 1D robot using CAD file and close-up photos

There are five structural modules (1_Pipette, 2_Back, 3_Front, 4_Top, 5_Trolley), the control brick module (6_Brick), and two optional modules (7_Sensor, 8_Gears) that make up the whole robot. We provide separate CAD files to construct these parts separately. Each main module requires about 30 minutes to build. To combine the syringe

with the Lego part, a red Lego peg included in the kit (Technic #32054 (*pin 3L with friction ridges lengthwise and a stop bush*)) can be glued to a syringe plunger. Cuvettes can be mounted on the 1D robot via double-side tape on the ruler.

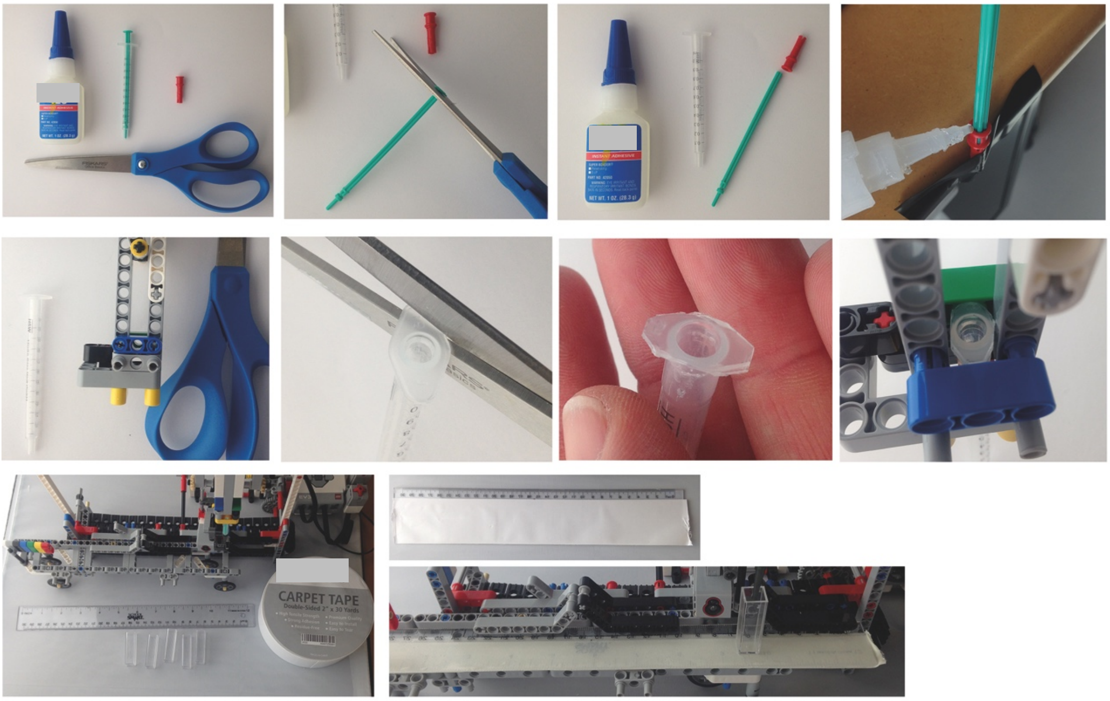

Fig S2. To attach the syringe's plunger to the robot, cut of the top of the green plunger, insert it into the red Lego piece and apply some instant glue. Cut away some of the plastic holding piece of the syringe's tube in order to fit it into the robot. Cuvettes can be easily placed onto a ruler using double-sided tape.

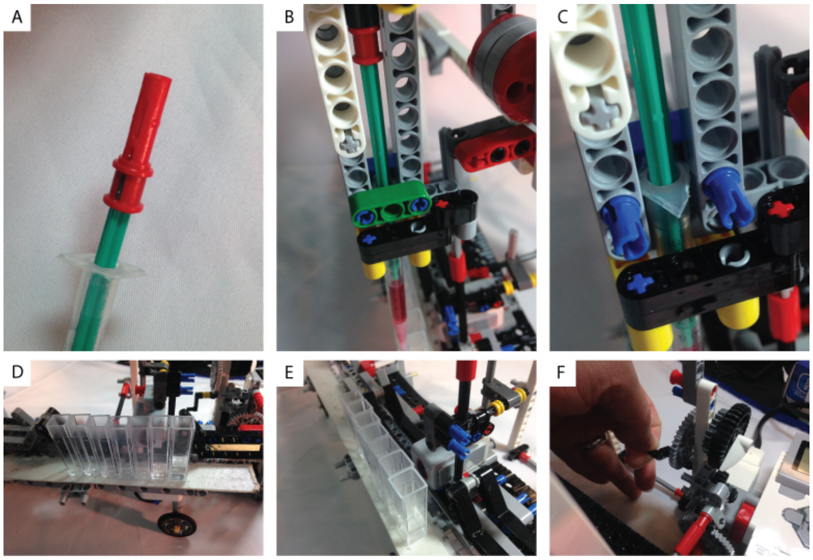

Fig S3. A) Prepared syringe with super glued red Lego piece and cut holder. B,C) Insert syringe while green piece is temporarily removed. Then reinstall the green piece to lock the syringe in place. Note that the lower part of the syringe has some wiggle room; if desired, this can be tightened up by placing some paper or cardboard

between the syringe and the yellow pieces in Fig.S3. D) Double-sided carpet tape on a plastic ruler allows securely placement of up to 20 cuvettes. Cuvettes can be replaced many times and even small liquid spills did not affect the tape's performance critically. E) A color sensor mounted behind the cuvettes allows to readout concentration and colors. F) Gears allow to manually moving the trolley over a crank.

The CAD file *1D_robot.lxf* contains the whole robot. This file gives an overview and shows how the different pieces come together. To build the robot, we recommend using the "Building mode" in the Lego software for each separate part (*1Pipette.lxf*, *2Back.lxf*, *3Front.lxf*, *4Top.lxf*, *5Trolley.lxf*, *6Brick.lxf*, *7Sensor.lxf*). In the building mode, it may be difficult to see the correct length of axles. Therefore table S1 below gives additional details for each CAD files when in *Building guide mode (F7)*:

Table S1. Axle length (L) guide for the individual parts of the 1D robot.

**1_Pipette**	Step 7, L=4	Step 8, L=3	Step 10, L=4	Step 11, L=3	Step 14, L=5				
**2_Back**	Step 8, L=9	Step 17, L=3							
**3_Front**	Step 7, L=8end	Step 11, L=7	Step 16, L=3	Step 23, L=5	Step 26, L=8	Step 28, L=8	Step 34, L=3	Step 36, L=8end	Step 37, L=4
**4_Top**	Step 4, L=5	Step 12, L=12	Step 14, L=12	Step 16, L=9					
**5_Trolley**	Step 1, L=5	Step 8, L=5	Step 10, L=6	Step 20, L=4					
**7_Sensor**	Step 1, L=8	Step 4, L=7							
**8_Gears**	Step 3, L=9	Step 8, L=7	Step 11, L=3	Step 13, L=3	Step 15, L=4end	Step 20, L=4end			

2.1.1. 1_Pipette module

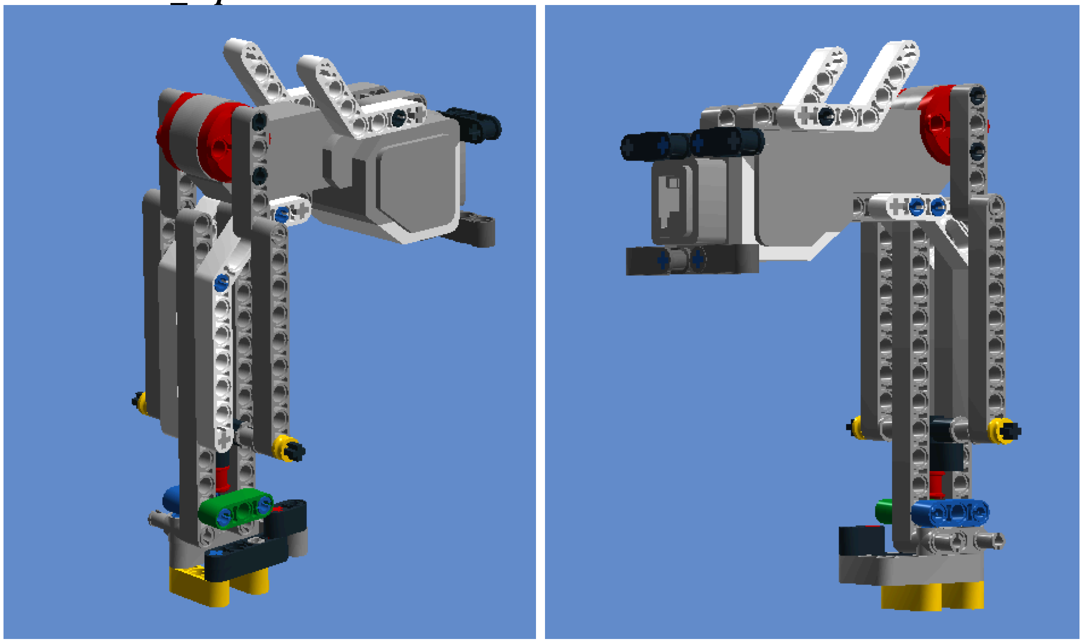

Fig S4. CAD representations of the pipette head module.

2.1.2. 2_Back module

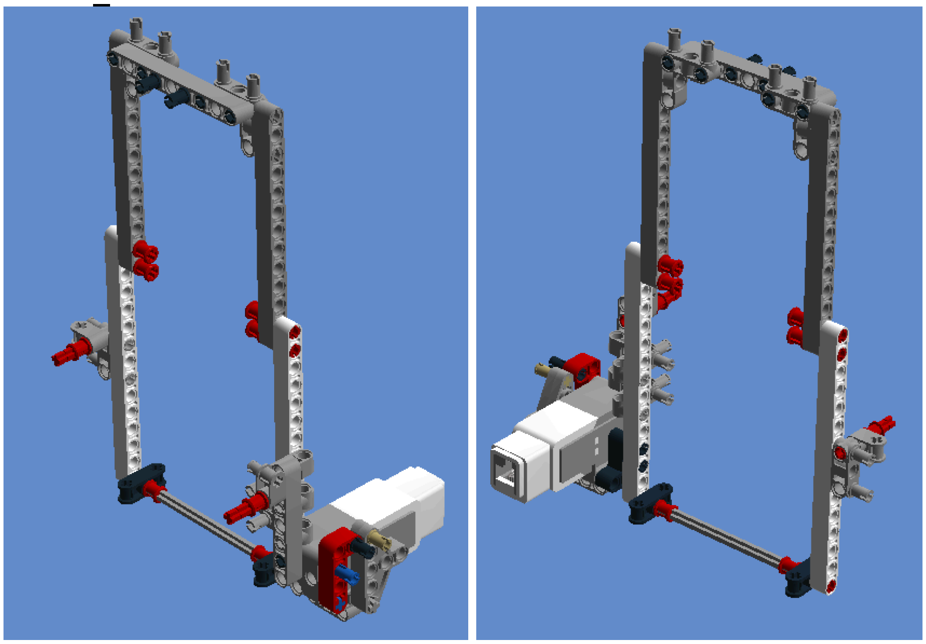

Fig S5. CAD representations of the back module without the gears.

2.1.3. 3_Front module

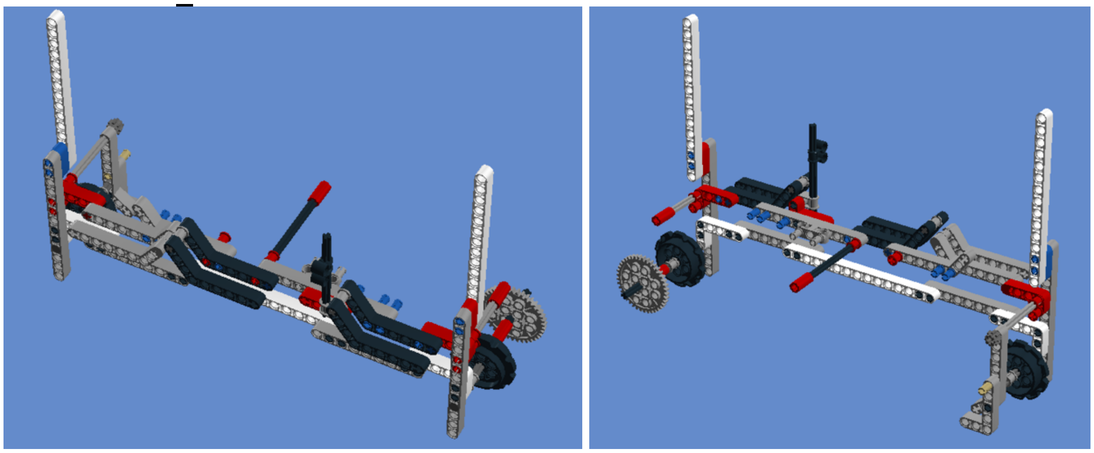

Fig S6. CAD representations of the front module of the 1D robot (without the belt that loops around the two black wheels.)

2.1.4. 4_Top module

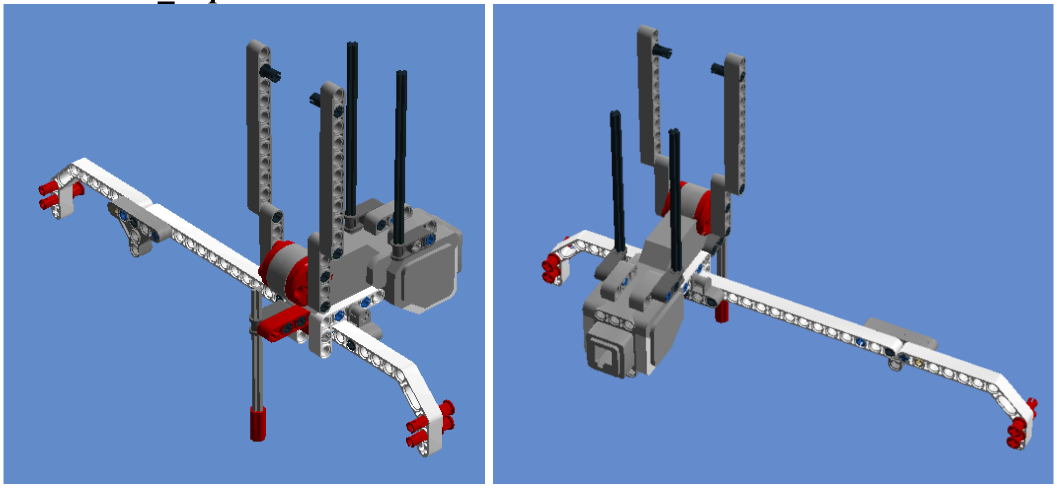

Fig S7. CAD representations of the top module.

2.1.5. 5_Trolley module

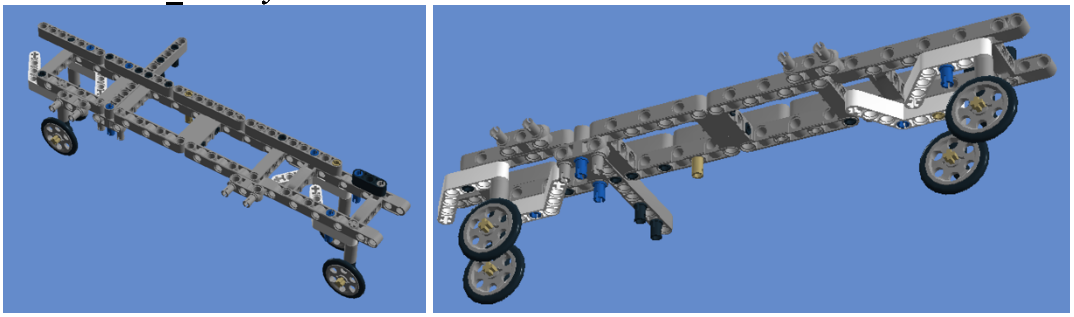

Fig S8. CAD representations of the trolley module.

2.1.6. 6_Brick module and *7_Sensor module*

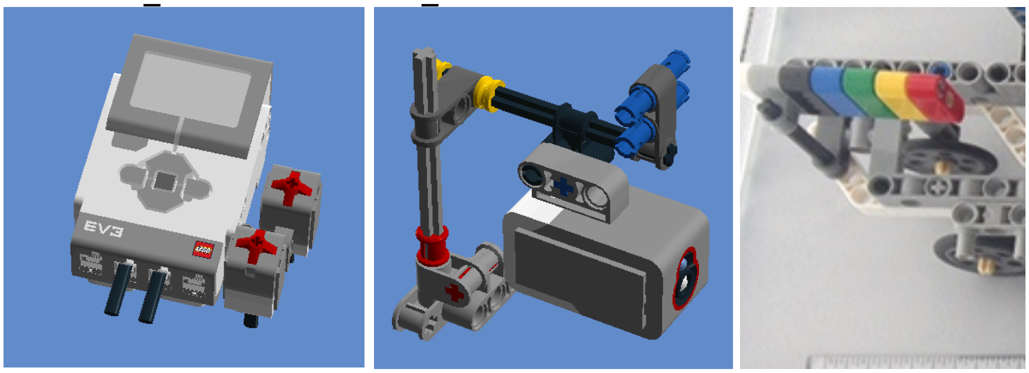

Fig S9. CAD representations of the control brick (left) and sensor modules (middle). Adding a colored bricks (right) or other colored markers on the trolley module or the samples allow for automated positioning and homing routines of the robot.

2.1.7. 8_Gears module

By adding some gear wheels (see CAD file *1D_8_Gears_(with_2_Back).lxf*) to the robot, the trolley could be operated manually by turning a crank. This also transfers mechanical knowledge to the user.

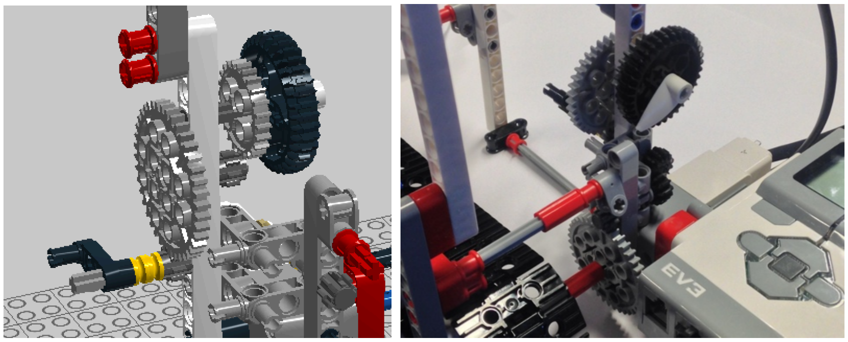

Fig S10. Gears module to manually move the trolley.

2.2. Performance of liquid dispensing and robot stability

The pipette is easiest operated via a 180 degree turn of the crankshaft motor that operates the syringe plunger (Fig. 1C-E main paper). For used the 1 ml pipette this translates to 720 µl (due to some design robot constraints the plunger does not travel the full 1 ml distance). Here the precision was 1%, and the accuracy 2%, which was determined by weighing the ejected volume multiple times, and comparing to the weight of 1 ml of a P1000 pipette; the accuracy is given by accuracy of the balance and the P1000. Operating with a 90 degree turn, half that volume can be administered. All of the following activities utilize either 720 or 360 µl volumes at a time.

Administering smaller volumes is possible but challenging: The non-linear relationship between motor turn and linear pipette advancements requires particular conversion and calibration when a sequence of droplets is administered. Furthermore, especially for smaller droplets (~50 µl or less) the speed of ejection matters also as droplets may not leave the tip for slow ejection speeds. Here a solution is to advance the whole pipette with the droplet still hanging at the tip onto the underlying liquid or hard surface, so that droplet then releases itself. With these approaches, drops as small as 7 µl (20% precision, 30% accuracy) can be delivered by first loading the full 720 µl into the pipette, then releasing half of that (i.e., turning the crankshaft motor by 90 degree), and then only advancing this motor by another 1 degree, then dipping the droplet onto a surface. To make another droplet of that size, the whole pipette is discharged first, and the whole procedure starts from the beginning. Precision and accuracy was determined by making multiple such drops, taking an image, and then measuring the relative cross area of each droplets by drawing a rectangular bounding box around it. In parallel, droplets of smaller, similar, and larger sizes were made with P2, P10, P100 pipettors; the resulting droplets led to a calibration curve for converting the apparent area into an actual volume. The variation in volume of the droplets made with the Lego pipettor enabled to estimate the precision; the uncertainties in this calibration curve enabled to estimate the accuracy.

If smaller liquid volumes and higher precision are desired, we strongly recommend to use the pipette design described in section 3 (see also Fig. 1F,G in the main paper) as it has a linear relationship between motor turns and plunger advancements, furthermore can eject droplets at much higher speeds.

To test the mechanical stability of the robot, we ran 1,000 loops of a pipetting routine. 20 cuvettes on a ruler were filled 50% with water. The program then made 1,000 loops of [lower pipette head / fill in liquid / lift pipette head up / move trolley by one cuvette width / eject the liquid]. While we were able to run 1,000 loops with no issues, we would like to mention a couple of weak points we are aware of: (i) If run from batteries the brick controlling and powering the motors may shut down unexpectedly. So for long unsupervised runs, we recommend using the power adapter included in every Lego set. (ii) Some Lego parts may get loose over time due to repeated movement. We observed that after a robot is "broken-in" (i.e. parts that fell out were securely put in place) no more problems occurred. (iii) The syringes we used are typically intended for single use (e.g. when used for flu shots). Therefore, they are prone to internal abrasion after 100s of actuations. Even if some of the syringes lasted 1,000+ runs, some got sticky and needed to be replaced after <1,000 runs. An experienced user is able to exchange the syringe within seconds. (iv) Insufficient glue to secure the syringe plunger to the red Lego peg may result in detachment and failing of the robot. Reinserting and applying additional glue will resolve this issue in most cases. (v) We observed some inexplicable shut-downs of the Lego Brick. Renewed uploading the program code from the computer resolved this issue.

2.3. Software files 1D robot

Use the *Lego Mindstorms EV3* software to open the file *1D.ev3*. It contains the following six functions (names in bold):

- **Manual control:** Allows users to control the robot by using the up/down buttons to lower/rise the pipette head, the left/right buttons on the brick to move the trolley left/right, and the two touch sensors attached to the brick to take up respectively release fluid (Fig. 1A).
- **Dilution series with mix:** A simple loop to perform a dilution series. First the pipette head is lowered (motor B turned 180°), then the cuvette is mixed (motor C turned 180° twice to fill and eject liquid into syringe,) then the syringe is filled (motor C turned 180°). After lifting the pipette head (motor B turned 180°), the trolley is moved to the next cuvette (motor A turned 38°) to finally release the liquid again (motor C turned 180°). This loop is repeated 5 times to obtain a dilution series similar as show in Fig. 2B.
- **Density layers:** Submerges a denser liquid below a less dense liquid (Fig. 2D).
- **Stress test loop:** A loop similar to the dilution series code shown above contains 20 dilution steps and is executed 50 times. This results in 1,000 or more operations of each lowering, filling, lifting, moving, and ejecting.
- **RBG readout:** RGB readout block from http://mindcuber.com/. RGB values are displayed on the brick. Best results are achieved if a white paper is place

on the other side of the cuvette. Results may vary as the readouts may vary and calibration might be required. See below for results to be expected.

- **Dilution series:** Same as above, but without the mixing step.

2.4. Experiments 1D robot

In the following we provide detailed protocols for all experiments so users can reproduce them. They are ranked easy, medium, or advanced difficulty.
When doing these experiments you may want to use the following items:

2.4.1. Fixing the cuvettes (easy, 5 min)

Use double-sided tape to place the cuvettes onto the ruler. The tape is strong enough while allowing exchanging the cuvettes countless times.

2.4.2. Manual control of the robot (easy, 5 min)

The file *1D.ev3 – Manual control* allows to control all three motors by the push of a button on the brick and with the two push sensors. This was used for the manual hand pipetting part in the user studies. The push buttons move the piston, left/right buttons on the brick move the trolley, and up/down moves the pipette head. Each speed and direction can be individually adjusted in the *Lego Mindstorms EV3 Home Edition*.

2.4.3. Programming of the robot (easy, 30 min)

The robots can be programed with the free Lego software *Lego Mindstorms EV3 Home Edition*. Building blocks can be drag-and-drop in order to program a code sequence. The program must be uploaded via USB, Bluetooth, or WiFi.

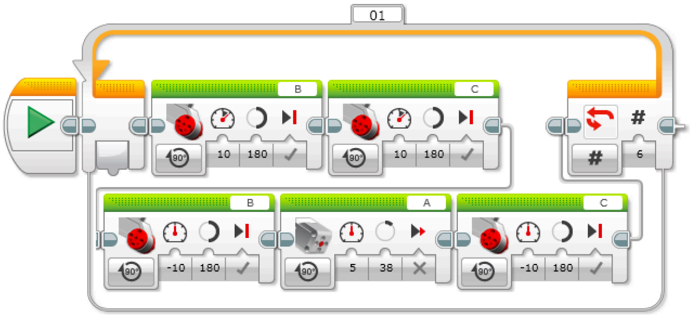

Fig S11. Screenshot of the Lego software. Here, the sequence for a dilution series is shown. (i) The pipette is lowered (motor B, 180°), (ii) take up liquid (motor C, 180°), (iii) lift pipette head (motor B, -180°), (iv) move trolley (motor A, 38°), (v) eject liquid (motor C, 180°)

2.4.4. Mixing experiment (easy, 5 min)

We used food color mixed with tap water. Typically, 3 drops per 50 ml water gives nice deep colors. Here, 2 loads (two times approx. 600 ul) were transferred each from the blue and the yellow cuvette into a third cuvette. The resulting solution will be green.

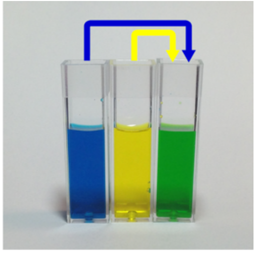

Fig S12. Color mixing.

2.4.5. Dilution series experiment (easy, 10 min)

Place a total of 7 empty cuvettes on the ruler. Make sure they stand close together and are aligned with the pipette tip. Fill the left cuvette with your favorite color (here blue) to about 80% full. Fill the other 6 cuvettes with water to about 60% full. Take a full load from the blue cuvette and transfer it in to the second cuvette. Then take a load from the second and put it in the third cuvette. Repeat. This can be done with pasture pipettes, with the pipette head only using the manual program, by the robot using manual control, or completely automated with a preprogrammed sequence. If the software is used to program the robot, speed, direction, and degrees turned might be adjusted.

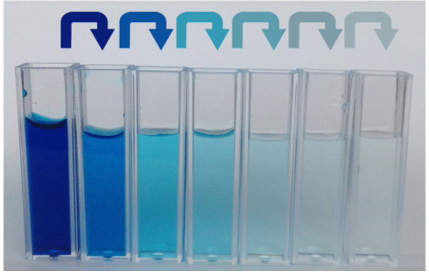

Fig S13. Dilution series.

2.4.6. Color readout (medium, 30-60 min)

Color and concentration readout are a bit tricky. The sensor must be placed as close as possible in front of a full cuvette. For best readouts, a white paper can be place directly on the other side of the cuvette. The values (best choose reflected light) can be readout in the software or on the brick directly. For RGB value we used the code provided in a separate file. Import ColorSensorRGB-v1.00.ev3b as a new block into the Lego Mindstorms EV3 software. Details from the creator can be found at http://mindcuber.com/mindcub3r/mindcub3r.html. After finding the RGB values, the color can be regenerated by a program such as Photoshop.

R-value	8	245	2
G-value	65	255	126
B-value	84	6	4
Color			

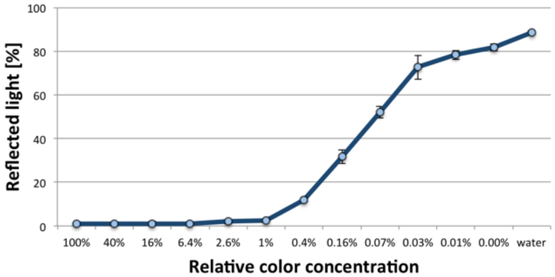

Fig S14. Color and intensity readouts.

2.4.7. Density layer experiment (medium, 30 min)

The salt solutions used for the density experiments were prepared by dissolving 18.0 g, 12.0 g, and 5.9 g sodium chloride in 50 ml water to obtain a saturated (100 %), 67 % saturated, and 33% saturated solution. Given the salt content, the solutions have different densities and can therefore be stacked on top of each other. Using a Pasteur pipette, starting with the densest (blue) and sequentially less dense solutions seems to be the easiest. If using the robot, submerging the least dense (yellow, water) with denser solutions is easier as less mixing occurs. Using the code provided, the user should therefore pipette the solutions in the order blue, green, red, yellow to obtain the best results. This experiment can be simplified by using only two different densities. In the user studies three groups made three layers and one team managed to make all four layers. In a quiz setting, the students can be asked to figure out which solution is denser if they are not told before.

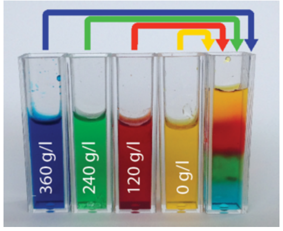

Fig S15. Salt density layers

2.4.8. pH indicator experiment (medium, extended preparation, 30 min)

To make the cabbage juice, 300 g of fresh red cabbage was blended with 300 ml in a NutriBullet food processor for 30 seconds. Subsequently, this mix was boiled on medium heat for 5 minutes. After letting sit to cool down to room temperature the mix was filtered through a standard coffee filter. This experiment also works with cabbage in glass or “red cabbage extract” kit (both available on Amazon). For each sample of 3 syringe loads (total 1.8 ml), 1 load of cabbage juice was added. The pH of each sample was measured independently with Fisher pH paper (Cat. No. 13-640-508) or reported as given by the producer.

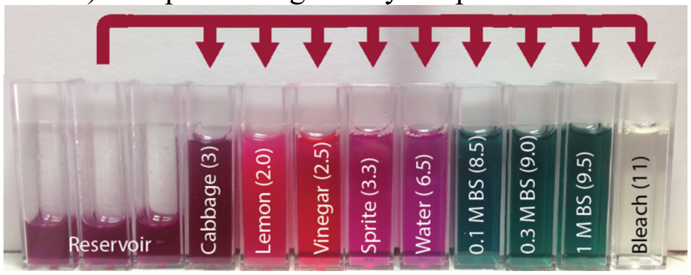

Fig S16. pH experiment.

2.4.9. Yeast growth (advanced, 60 min)

We used Red Star Active Dry Yeast. 7 g (1 packet) were suspended in 35 ml tap water. After intense mixing for 2 minutes, this suspension was added to the sugar solutions. The sugar solutions were obtained through an automated dilution series. Standard household sugar was used (granulated cane sugar).

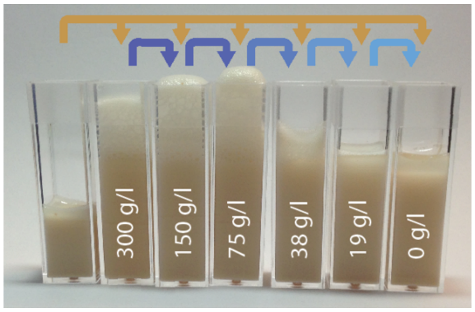

Fig S17. Yeast growth at different sugar concentrations.

2.4.10. Disinfection from E.coli (advanced, special equipment required, 30 min)

To show that the pipette can be used to work with bacteria without the need to change the tip or the whole syringe, we dipped the pipette into 10% bleach. E.coli (1st cuvette) contains an overnight culture of E.coli in LB medium. The concentration is a bout 10^8 colony forming units / ml. If the pipette is used to suck up and eject pure LB medium after the E.coli suspension was transferred with the pipette, new bacteria will grow in the second cuvette. Therefore the previously clear LB medium became opaque (2nd cuvette). But if the pipette is used to suck up and eject pure 10% bleach (3rd cuvette) after the E.coli suspension was transferred with the pipette, the pipette can be used again to transfer fresh LB medium without any new bacteria growing. Therefore the clear LB medium remains clear (4th cuvette). This also works with 70% ethanol, but not with vodka (40% ethanol).

Please note that E.coli should be handled with caution and according to corresponding instructions. They can be obtained e.g. from Carolina (http://www.carolina.com/bacteria/escherichia-coli-living-k-12-strain-plate/155067.pr).

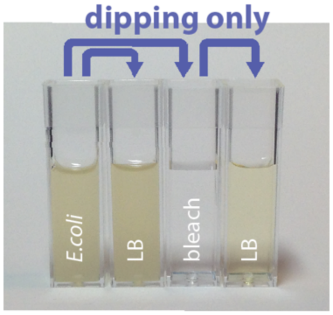

Fig S18. E.coli growth and disinfection of the pipette using bleach.

2.4.11. Skipping cuvettes (easy, 10 min)

Complex mixing patterns may emerge if a two code routines (such as a dilution series) are overlaid. Here, we first made a dilution series of the blue solution while every second cuvette was skipped. Then a second dilution series was made starting at the orange cuvette while skipping always two cuvettes.

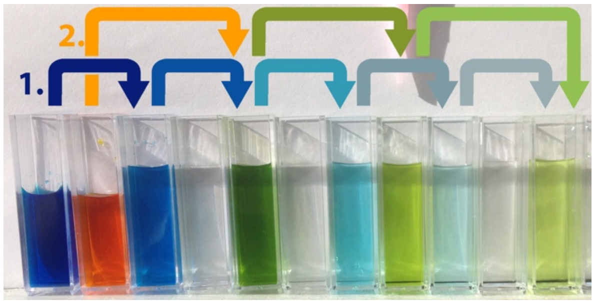

Fig S19. Emerging patterns due to programming routines.

2.4.12. Smaller drops (advanced, 60 min)

In order to deliver volumes of liquid smaller than a full load (approx. 800 microliter) the motor can be turned for less than 180°. As the motor rotates and the syringe is linear, the ejected amount follows theoretically a sinusoidal behavior. However, since often some air remains in the syringe the first 90° and the last 90° do not result in the exact same amount. This non-linear behavior can be explored and calibrated. For example: If one turns the motor for 90° (approx. 400 microliter), then dips the tip onto the underlying plastic surface to release all liquid from the plastic tip, the next drop (e.g. 10° turned) always results in a very similar ejected volume.

3. 2D robot - Building plan, instructions, and experiments

We fitted a linear pipette head onto a robot that can access both the x- and y-axis. At the cost of higher complexity and additional parts (two additional motors are needed and therefore this robot requires four motors in total) this 2D robot featuring the linear slider-crank pipette head is able to address up to up to four 96-well plates (i.e. +-2 mm spatial precision) and reliably deliver volumes as small as 1 ul to up to 384 defined wells. Two touch sensors and one color sensor are used to home in all four dimensions (x, y, z, and fill level) and to allow for feedback-loops, ultimately allowing to run complex automated programs such as dilution series and color mixing in 96-well plates.

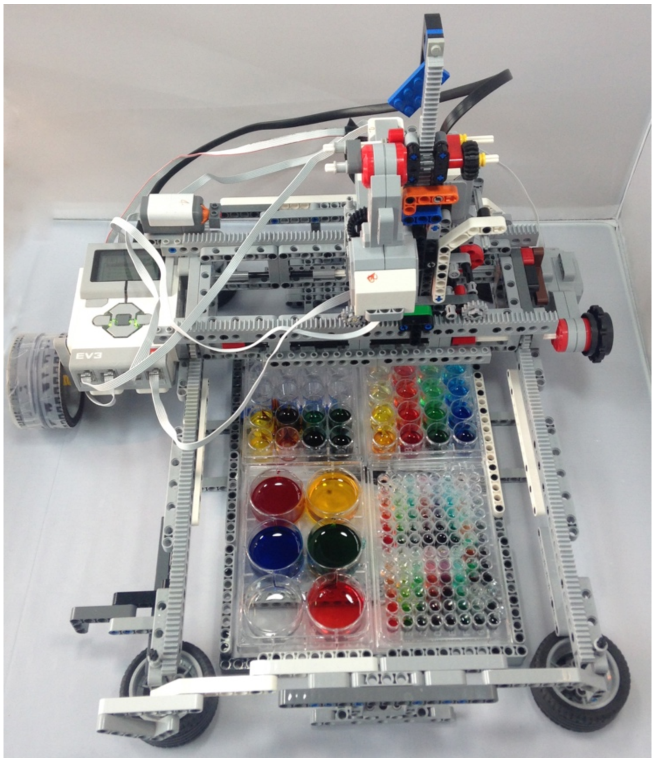

Fig S20. 2D robot with completed dilution series and mixing into a 96-well plate.

All required Lego parts are listed in the file *2D_Part_list.xlsx*. In addition to the parts mentioned for the 1D robot above (syringe, glue, food color) here well plates (6-, 24-, and 96-well plates (Amazon #B0177QVE1S, #B0177QVILY, and #B0177QVE7C, respectively)) can be used.

3.1. Building 2D robot using CAD file and close-up photos

The 2D robot requires more parts than the 1D robot and is significantly more complex. The supporting file *2D_Part_list.xlsx* list all Lego parts needed to build the 2D robot as presented here. Lego novices are encouraged to build the 1D robot before building the 2D robot.

The CAD instructions for the complete 2D robot is split into 5 separate files. These 5 parts should be built separate and then joined together while inserting the syringe.

Similarly to the 1D robot, the syringe is slightly modified (cut off green top and trim side holders) to glue it to a black Lego piece and put in place.

3.1.1. Syringe

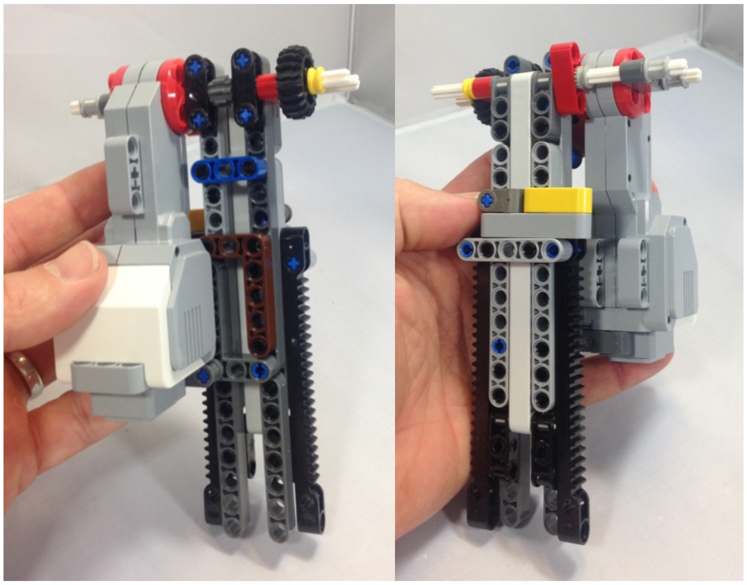

Fig S21. Linear pipette head module.

3.1.2. Lift

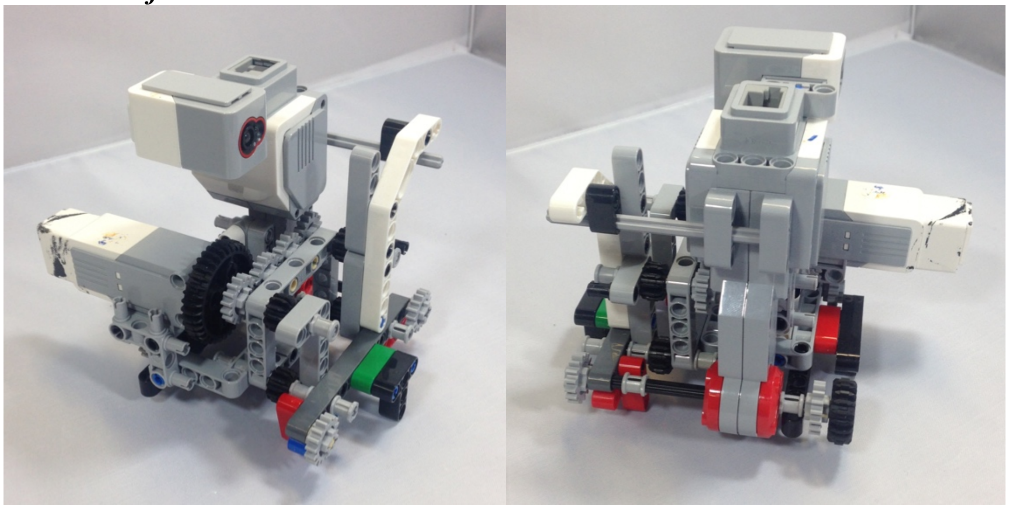

Fig S22. Pipette head lifting module with color sensor for orientation.

3.1.3. Prepare the syringe with blue "light sensor flag"

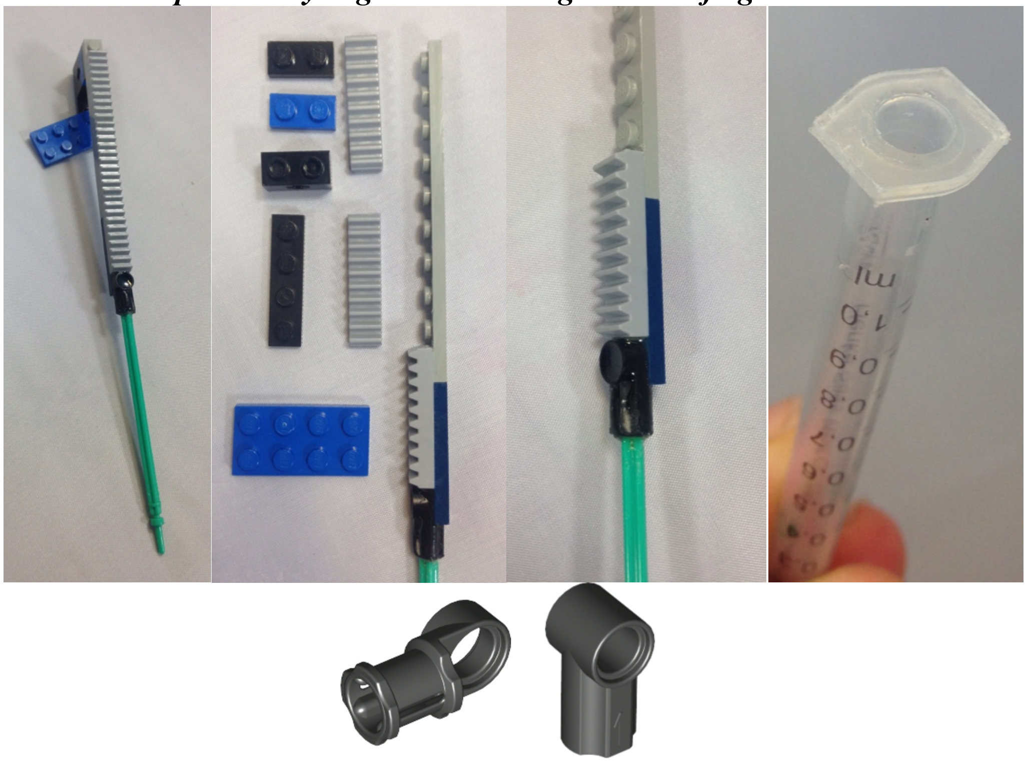

Fig S23. Preparations of the syringe for the 2D robot.

Ideally the piece on the left (4660886: Toggel Joint) can be used to link the plunger to the robot. Alternatively, the piece on the right (4107085: Angle Element, 0 Degrees [1]) can be used. There is the need to cut parts of the plastic away until it fits as shown here. Super glue is used to secure the connection.

3.1.4. Add the syringe

Cut some parts of the plastic top of the syringe to be able to fit it into the robot.

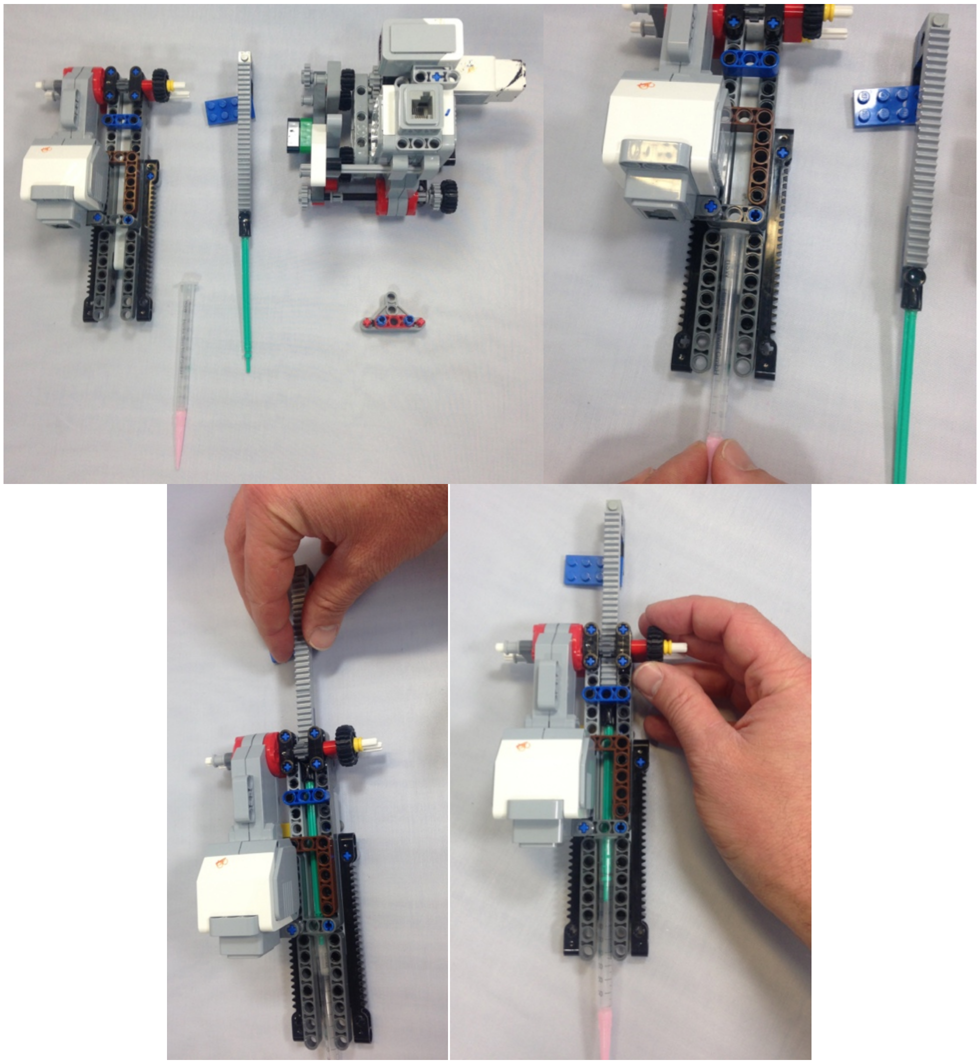

Fig S24. Inserting the syringe into the pipette head.

3.1.5. Merge Syringe and Lift parts including the syringe

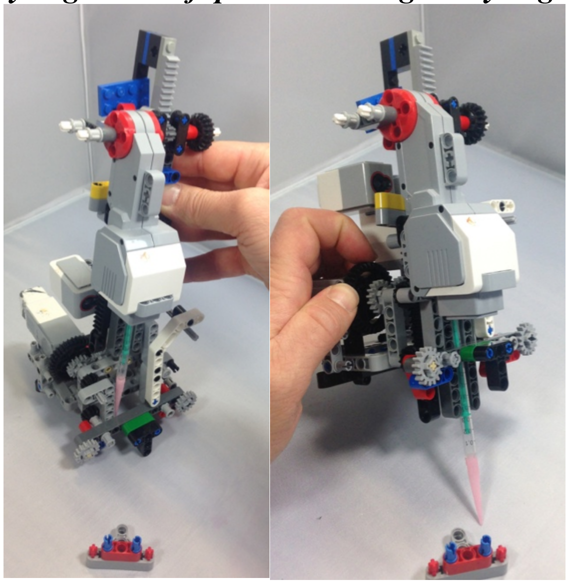

Fig S25. Inserting the pipette head into the lifting unit.

3.1.6. Cap

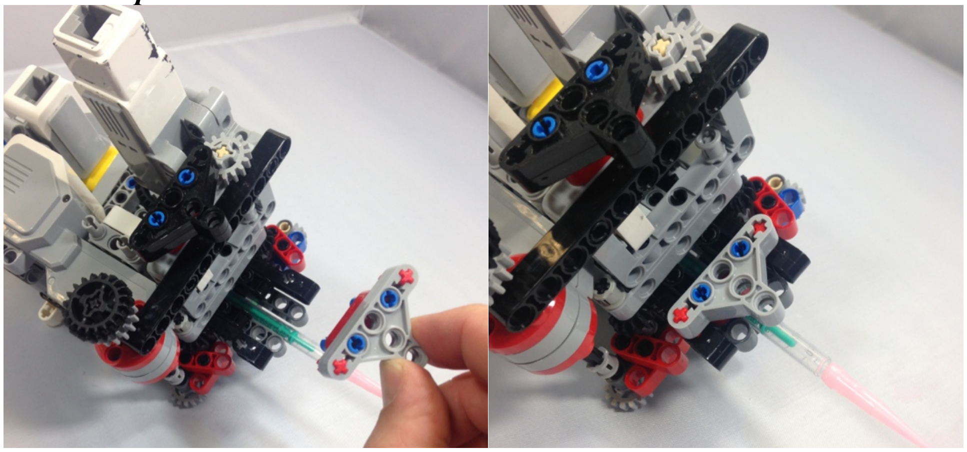

Fig S26. Locking the syringe in place.

3.1.7. Frame

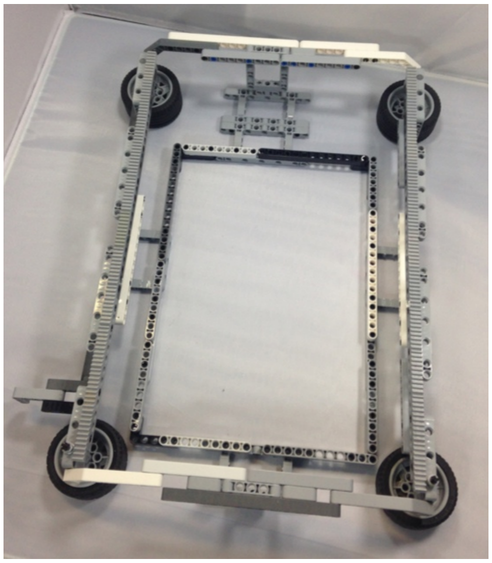

Fig S27. Base frame for x-direction movement.

3.1.8. Cart

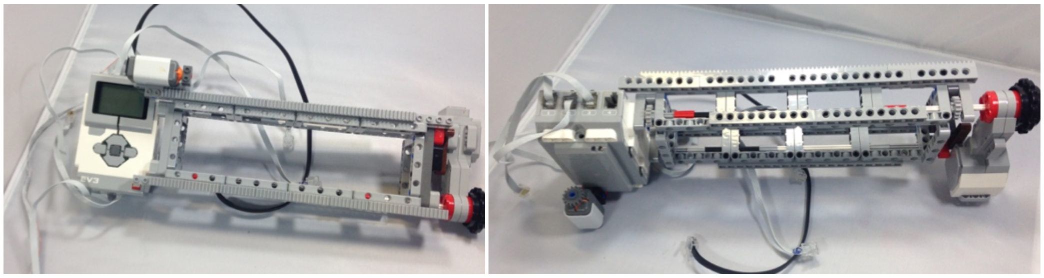

Fig S28. Cart with controller brick for y-direction movement.

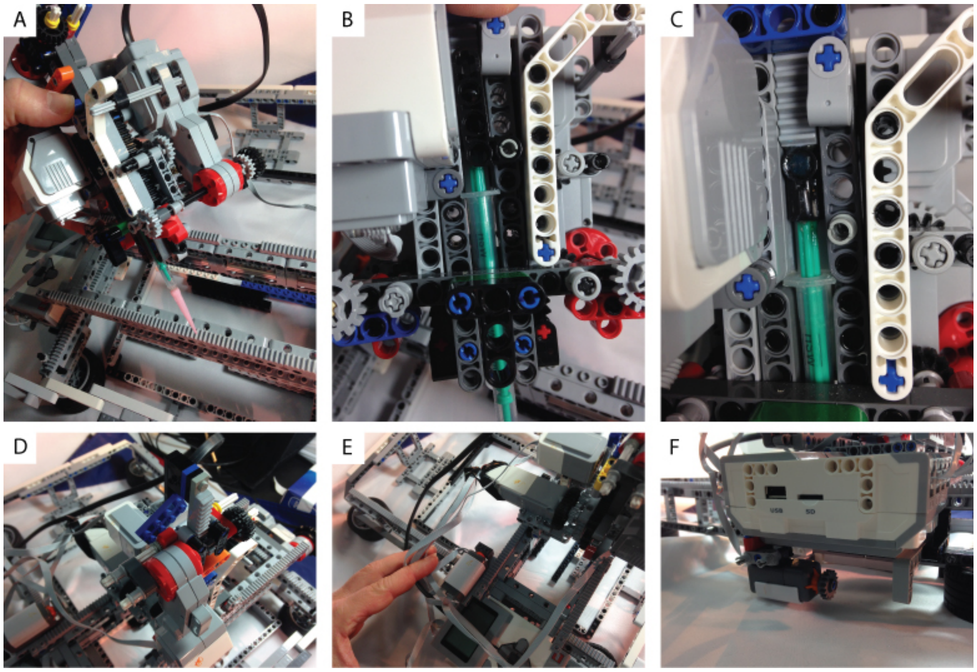

Fig S29. A) The pipette head sits on a rail system and can be lifted up easily. B) The syringe is mounted on a railed system to allow linear actuation. C) The pipette's plunger was super glued into a standard Lego piece that connects to the robot. D) A light sensor is used to reset both the fill level of the syringe as well as the z-position of the pipette head. (See 2D.ev3 code file where the ClearAll function cleans the pipette and resets all motors to a starting position). E,F) Two touch sensors are used in to reset the robot in the x and y direction.

3.2. Software files 2D robot

The 2D robot can be programmed analog to the 1D robot using the *Lego Mindstorms EV3 Home Edition* software. We provide here the file 2D.ev3 to perform a serial dilution in a 24-well plate positioned as 1G and movie S2. The main block *Row1_Dilution* will reset all four motors and then perform a serial dilution into prefilled wells by using the following blocks:

Row1_Dilution
- *ClearAll* → reset Z position / empty and wash syringe / call X and Y reset
  - *XandY_Reset* → reset X and Y position
- *Full_Color_1stDilution*
  - *Dilution_Take_In_FullColor* → take up liquid
  - *Full_Color_dispenser* → eject liquid
  - *SolutionMixer* → mix liquid in new well
- 4x *Basic_Dilution_AndMixing* → take up, move, eject, mix
  - *SolutionMixer*

More complex operations can be programmed. However, with increasing complexity – e.g. if addressing multiple 96-well plates – the program becomes significantly slower.

3.3. Experiments 2D robot

3.3.1. Analog to 1D

We successfully performed similar experiments as with the 1D robot (i.e. color mixing, dilution series, density layers, yeast growth, sterilization, color readout) and expect that – given its precision both in x/y position and volume control – such complex robots could be used and adapted to make real biotech experiments.

3.3.2. Standard lab ware: 6-, 24-, and 96 well-plates

The 2D robot is able to address standard well-plates and measure and transfer volumes as small as 1 microliter. The figure below shows how each color (green, blue, yellow, red) are diluted in an automated dilution series in a 24-well-plate. Then these are mix into a 96-well-plate at various ratios as indicated in the schematic. The well labeled with "Trash" is used to eject unused solutions into. The well labeled "Wash" is used to flush the syringe (with what is currently in that well at the start and ideally this is water).

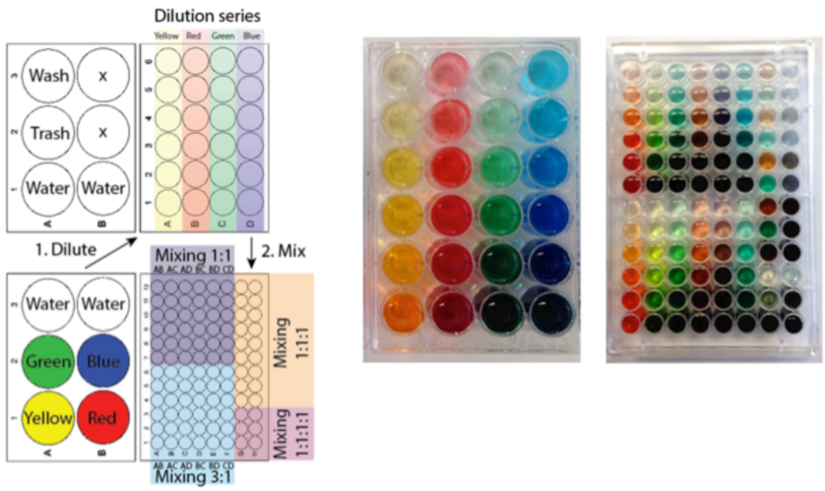

Fig S30. Schematic of programmed dilution and mixing patterns and resulting 24- and 96-well plates.

3.3.3. Modes and performance of liquid delivery

The 2D pipette tip can deliver drops below 1 microliter (Fig. 1H,I, main paper). Unlike the 1D robot described above, this 2D robot features a pipette head that is actuated in a linear fashion via a gear track (Fig. S24). This makes the delivered volume proportional to the degree motor rotation, i.e., different volumes can be delivered easily (Fig. S31). A major determining factor is the diameter of the syringe plunger, where a smaller syringe enables smaller droplets. In the following we first discuss the usage of a simple and low cost 1 ml plastic syringe, and then the usage of a more expensive 0.025 ml Hamilton syringe.

Two modes of operation were tested for the 1 ml syringe: *Ejection mode* shoots out the fluid a high speed to detach all fluid immediately from the tip. *Dip-in mode* (Fig. S31) releases the fluids slowly, and subsequently lowers the tip to touch the surface to ensure detachment of all dispersed fluid. In either case, the pipette should be advanced in multiples of 1 degree as that is smallest resolution that the Lego motor encoders have.

The Ejection mode has the advantage that the pipette does not come into contact with another medium thereby avoiding potential contamination. The ejection mode typically should only be considered for droplet volumes that are larger than 50 µl – since a certain speed is needed for the drop to release from the tip of the needle. Although smaller droplets do release, sometimes the size from drop to drop varies as smaller drops remain at the tip of the needle. This can lead to effects, for example, that advancing the motor by 10 degree (about 10 µl – see calibration below) can lead to very reliable droplets, while 9 and 11 degree do not. Determining factors are: properties of fluid (we only tested liquids with properties similar to water), the pipette tip opening size and properties, the speed of ejection (if the speed is too low, the droplet may release from the needle tip; if the speed is too high the droplet may break-up into multiple ones), the specific drivers that are

used (e.g., the EV3 brick, the NXT brick, or third party controller all can slightly differ in their motor actuation characteristics and consequently affect the droplet release). We recommend to systematically try all parameters available, typically high speeds and small pipette opening diameters are advantageous. The droplets themselves can be ejected at a rate of ~1/sec.

In the dip mode much smaller droplets can be delivered reliably, where after releasing the liquid, the pipette head is lowered until the drop (or the pipette directly) touches the underlying surface or liquid. This has the drawback of potential contamination of the fluid inside the pipette. The maximum droplet delivery rate here is slightly slower, i.e., ~ 3-5 sec.

The accuracy of the pipettor was determined by loading the syringe with fluid, then advancing the Lego motor by 360 degree or more, and then weighing the dispensed liquid amount, and setting this in relationship to the advanced degrees. This mass is also calibrated against 1000 μl water dispensed from a P1000 pipette, which gave 990 mg. The accuracy of this calibration is then limited by (1) the accuracy of the P1000 (1.2% according to ISO 8655); (2) the accuracy of the used balance (0.1 mg, which is negligible for liquid volumes of ~0.5 ml, i.e. ~500 mg); and (3) the uncertainty in the mean droplet volume ejected from Lego pipette, i.e., the standard error of the mean of the measured mass (when using pragmatically only 2 tries, this leads to 6% uncertainty, but this uncertainty can be made arbitrarily small by dispensing and weighing liquids many times over and using the standard error of the mean as estimator; in order to bring this value below the other contributions, i.e., significantly below 0.5%, this would require over 100 measurements). Taken all this together, we can calibrate the 1 ml syringe to 1.24 μl/degree at an accuracy of 8% (although accuracies down to 2% might be achievable).

The precision of droplet volume was assessed by placing 6-10 droplets on a plastic surface, taking images of these droplets, measuring their apparent area (by drawing a square bounding box around the drop, for example using imageJ), and then converting this value to an apparent volume by taking it to the 3/2 power. (Note 1: For droplets of vastly different sizes this area to volume conversion introduces systematic errors as bigger droplets are flatter while smaller droplets are more rounded up. But this can be safely neglected for volumes within a factor of 2 as we tested by making droplets with standard laboratory pipettors P2, P10, P200. Note 2: One might also consider weighing individual droplets in order to determine the precision, but the required resolution and precision of the balance would be rather high, hence the procedure described here is much more straight forward.) The precision is then defined as the ratio of the standard deviation of the apparent volume and the mean of this apparent volume. This precision is likely also an overestimate, as variability in droplet shape (such as not being perfectly round) lead larger apparent variability in volume, while the true variability might actually be lower. We also followed the same procedure to determine the precision of standard laboratory pipettes to validate our method, and to obtain a

direct comparison between the Lego pipettes and standard lab equipment. Using these procedures, we then find: For 5-degree motor advancements, i.e., 6.2 µl, we measured a precision of 19%, while for 5 µl drops using a P10 pipette we measured 3% precision. For 2-degree motor advancements, i.e., 2.5 µl, we measured a precision of 26%, while for 2 µl drops using a P2 pipette we measured 18% precision.

In order deliver much smaller volumes, we tested a 0.025 ml Hamilton syringe, which has a glass cylinder and a metal plunger. We do not know the exact model number of the used syringe as we used one readily available in the lab, but it is equivalent to #80200 (http://www.hamiltoncompany.com/; 25 µL, Model 1702 N SYR, Cemented NDL, 22s ga, 2 in, point style 2; current list price: $40). This model can be fixed into the same Lego pipette head (Fig. S24), although the Lego pieces are slightly bent. Using this pipettor in dipping mode, we were able to generate droplets down to 0.15 µl. Here the precision was 15% and the accuracy 8% (or the accuracy could be brought down to 2% with more effort) - determined the same way as described above. In comparison, we tested a P2 standard pipette at 0.5 µl and found a precision of 47% (and an expected accuracy of 16% according ISO 8655). Note that repeated usage of small droplets with the Hamilton syringe would enable very precise volumes in the lower µl range, i.e., significantly improving the performance achieved with the 1 ml plastic syringe described above.

Overall, we conclude reaching the Lego pipettor can reach performance characteristics comparable to professional pipettors. There is a variety of contributions to this performance as discussed above, with the diameter and material of the syringe being a major factor. We recommend everyone in need of such high performance to systematically evaluate the particular system at hand and to calibrate accordingly.

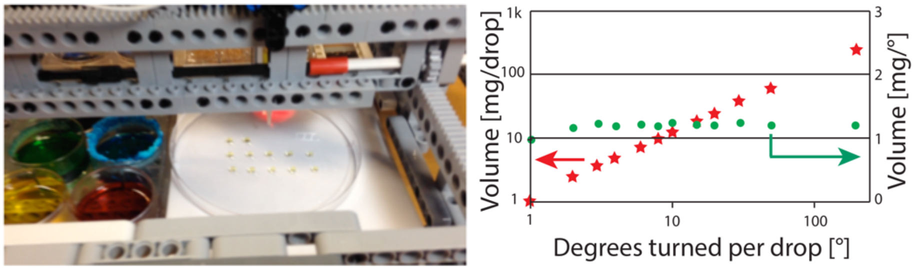

Fig S31: Dip-in mode of the 2D robot.

4. Example lesson plan (as deployed in elementary school)

4.1. Lessons plan

The building and experiments can be split into five 90-minute sessions as we did with the girl scouts test group (5th grade elementary school). See also main paper and chapter 7.2 for details.

.

Table S2. Session overview

Session	Activities	Results	Comments
**1. Building pipette**	• Introduction<br>• Pasteur pipetting<br>• Build pipette head	• Each team built a pipetting head	• Building time: 60 min<br>• Printed instructions
**2. Hand pipetting**	• Finish pipette head<br>• Experiments with hand-held pipette head	• Introduction<br>• All made all 3 exp.<br>• Self activated color on paper experiments<br>• Many repeated exp. with other colors<br>• Up to 3 layers	• CAD instructions<br>• Girls liked colors<br>• Teams of 2 work well for experiments<br>• 1 team used mostly Pasteur pipette
**3. Building full robot**	• Build all modules of robot<br>• Dry-run full robot	• Each team built 2 additional parts	• Time to complete robot (w/o head): 120 min / team
**4. Robot experiments**	• Experiments with robot	• All made all 3 exp.<br>• Some learning on density observed	• Advantages of robots: stronger / more controlled / help us / can move without stopping / can handle<br>• Disadvantages of robots: glitches / can break / can't talk / you have to fix it
**5. Programming**	• Programming<br>• Automated experiments	• 1 team (2/6 students) completed worksheet.<br>• 6/6 were able to control all 3 motors<br>• 6/6 students understood speed.<br>• 4/6 understood direction changes.<br>• 4/6 made a dilution series.<br>• 1 team (2/6) did not attempt to use liquids.	• 3/6 have programmed Lego before. 2/6 have not. 1/6 no answer.<br>• They incorporated sound effects.<br>• One student absent.

Table S2: Sessions as conducted with the girls scouts test group.

4.2. Example worksheets

See section S7.

5. Other robot designs

As described in section 6.1, multiple pipetting robots were designed through an iterative process, eventually leading to the two designs featured in figure 1 (main paper). We showcase three prototypes here, all of which differ in regard of how the sample vs. the pipette are moved. These examples demonstrate the general design freedom for more open-ended projects.

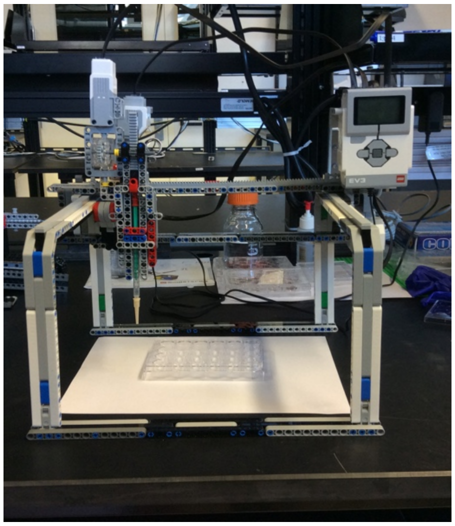

Fig S32. 2D robot with a stationary sample while the pipette head moves in x, y, and z. This design is conceptually equivalent to the robot featured in figure 2F,G in the main paper.

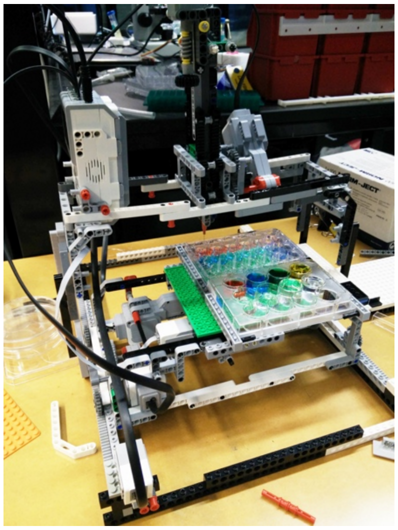

Fig S33. Robot with a stationary pipette head that could be lifted and lowered in z, while the sample could be moved on a x, y table.

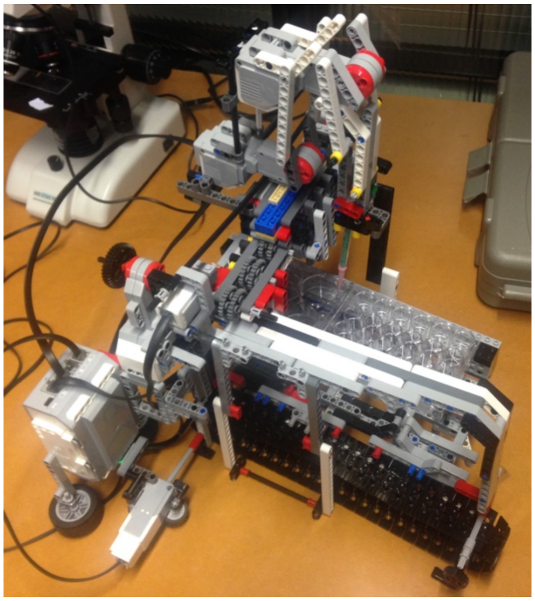

Fig S34. Extension based on the 1D robot featured in figure 1A in the main paper. An additional motor allows to access x and y axis. This comes at the cost of higher complexity and more parts (incl. an additional motor) and cannot be built from the EV3 kit alone. In this case the pipette moves in y and z, while the sample moves x, which constitutes a hybrid of the two previous designs.

6. Description and outcomes of the user studies

Working with students from elementary, middle, and high school, we tested three use cases: (1) Three high-school students (16-18 years old) working over the summer (between 4 and 8 weeks) in our lab developing their own robots as well as exploring STEM experiments that could be done on these robots. (2) Eight elementary grade students (10-11 years old) over five 90-minute after-school sessions, instructed by two researchers. (3) Twelve middle-school students (11-13 years old) over 16 sessions (about 30 hours total), instructed by one middle-school science teacher. While the total number of participants is small (~20), these activities reflect typical student numbers in after-school robotics classes. Our results provide sufficient insight and confidence regarding potential lesson plans, learning outcomes, and logistics, and how teachers could deploy and adapt these activities for their own students in the future. IRB is described in methods of the main paper.

6.1. First use case: High school students summer research project

Participant selection: Three students (both genders) approached us for summer research opportunities (4-8 weeks); they were between 16 and 18 years old, and all had prior experience with Lego Mindstorms. Additionally, one teacher was present in the lab during this summer as well as part of the IISME program at Stanford University (Industry Initiative for Science and Math Education). This teacher worked with the students, and then also later used one of the robots with her own robotics class (see use case #3). All three students and the teacher are co-authors on this paper.

The general approach was to let the students and teacher (in frequent discussion with the researchers) develop their own robots as well as explore and develop STEM experiments that could be done on these robots and that would be suitable for certain target audiences and align with curricular needs, especially regarding the Next Generation Science Standards. As a motivating starting point a pipetting robot was used that had been previously developed and deployed for a remote experimentation lab (Hossain et. al 2015, see main paper for detail). This robot had been built in part from Lego, but also included a number of "hacks" such as controlling the whole robot from a Raspberry Pi computer, integrating a flat-bed scanner for image acquisition, and many soldered connections. Hence this robot was hard to replicate without extensive engineering experience, though it had demonstrated that reliable liquid handling with Lego is possible in principle. We intended to bring this general concept to a level that would be amenable to a much wider audience.

We therefore defined the design goal to develop a Lego pipetting robot that could eventually be used in a school or afterschool setting at the middle school level.

The main designs attempted were (1) to move the pipette head in x, y, z while keeping the samples still; (2) to move the pipette only in z while moving the sample in x, y; (3)

move the head in x, z, while moving the sample in y. (See also chapter 6 for examples on each of these design options). Eventually we also considered the simplification of using only one degree of freedom to move the sample, which lead to the 1D robot in the paper; this design was further driven by the consideration that only three motors are available in the standard EV3 kit (and one motor is always needed for the pipette actuation).

Evaluating the various design attempts, several lessons were learned: We found that that the pipette head tended toward larger designs (in order to deliver reliable droplets). To mount this pipette head then onto three motorized degrees of freedom (where typically every additional degree of freedom needs to carry the machinery for the previous ones) can become rather clunky (additional complications arrive when including sensor for homing positions). These design iterations led to different pipette designs that differed both in their complexity as well as speed, volume, and reproducibility of being able to deliver liquids, two of which were then used in each of the two robots presented in the main paper. Other design lessons were that the color sensor did not work as easily as hoped to detect concentrations of colors (and other reagents) inside containers. We also found that the programs for the 2D robot can become rather cumbersome and big. Hence operation via external interfaces (such as a via Raspberry Pi – see http://www.mindsensors.com/ for examples) would allow in the future more openness in programming, and would also allow to include more suitable sensors. Also, the students had nearly unlimited supply in Lego parts, which again may have led to rather large designs; in contrast, when confining the goal to using the parts of one EV3 kit alone, the resulting robot was significantly lighter and also more effective in the tasks it could do.

Ultimately, these iterative designs and the insights gained led to the two robot designs presented in the main paper, which we consider to have achieved two target goals: (1) A robot that is minimal and entry level, also using only parts available in the standard educational set (Fig. 1A main paper); and (2) a robot that has 2D control and therefore allows rather complex experimentation (Fig. 1G main paper). Also, the idea of a hand-held module emerged (Figs. 1E,F main paper). Additionally, a number of science experiments were successfully developed during this summer (Fig.2 main paper). Finalizing the robot designs and STEM experiments as presented in the paper (und which were also used for the two following use cases studies) had the researchers involved much more directly.

Overall, the iterative building as well as programming and exploring science experiments kept theses high-school students busy and interested for a long time, i.e., these Lego liquid handling projects lend themselves for more complex and open ended robotics projects, e.g., at the high school level.

6.2. Second use case: Elementary school 5th grade Girl Scouts

Eight elementary-school students (5th grade) of the same Girl Scouts troop (10-11 years old; all female; only the data from seven is reported in this study according as one student did not provide assent – but this child participated in all activities; 4/7 had previous Lego Mindstorms experience) built and used the 1D Lego robot during five 90-minute long

after school sessions taking place about bi-weekly. Two researchers familiar with this project served as instructors and also took notes during the study. Participants were recruited as one instructor was the parent of one these girls, and all girls were in the same Girl Scout troop. Each of the five sessions was accompanied by a worksheet (see section S7). Students always worked in pairs, on two days one student was absent, in which case the other student worked alone.

The goals of this user study were to test whether these activities would be suitable for 5th graders. Specifically: (i) Test whether the liquid handling robot (i.e., each of its modules) could be successfully built by this age group and whether instruction materials was complete and appropriate. (ii) Test whether the students could execute different liquid handling experiments with different modes of pipetting (Pasteur pipette, lab pipette, robot manual, programmed robot), and whether these different modes would synergistically support each other. (iii) Test whether these activities encourage soft learning such as "Why is pipetting useful?" and "What are robots good for?" and what the students' opinion is about these activities in general.

Data collection was done by instructors taking notes about relevant student behavior, and letting the students fill out work sheets.

The study was run in five sessions, each about 90 minutes in length (see also main paper). The worksheets are provided in chapter 8 of these supplements. See also chapter 4 for session overview and outcomes.

Session 1: Building and manual pipetting
The students used Pasteur plastic pipettes to transfer and mix water colored with food color. Students then in pairs built the pipette head module and also electronically moved the pipette; for some groups a few pieces were still missing at the end of the session (which were added at the start of session 2). Instructions were provided as printed hand-out as generated by the Lego Designer, which led to some challenges in recognizing certain parts and how to attach them; instructors provided occasional help. For all following sessions we provided each student pair with a computer where they could follow the instructions directly from the design program, which worked much more efficiently, especially as students could virtually rotate and zoom the design.

Session 2: Experiments with pipette head only
We instructed students be careful attention regarding liquids and electronics, but this never became an issue for any of the sessions, i.e., the electronics always stayed intact. The students used the pipette head they had built to transfer liquids. Typically, one student would hold the pipette and another would push the buttons to suck up and release the fluids (see Fig. 1F main paper). Here working in pairs worked particularly well. The experiments they did were mixing colored solutions, dilution series of colored solutions, and salt gradient layer experiment (Figs. 2A,B,D in main paper). The usage of colored solutions seemed to be particularly attractive and motivating, as students repeated the experiments with their "favorite colors" on their own initiative (instead of moving directly on to the next experiment as they were instructed.) While three groups used the

Lego pipette module throughout, one group eventually did most of the experiments with the normal plastic pipette instead of using the robot pipetting head. One group successfully executed the three layer experiment (Fig. 2D in main paper) (other students did in later sessions). Multiple students started to also make colorful patterns on tissues (which we had originally provide to clean up spills), which again indicates that students liked working with colored liquids.

Session 3: Building complete robot
Every group (2 students each) built one or two modules for the robot. Depending on module and student group it took between 20 and 30 minutes for each of the modules to assemble. Besides occasionally minor mistakes that the instructors helped to fix, the students were successful in finishing all these modules. Students seemed to be faster compared to session 1, which we attribute in part to the digital (rather than printed) instructions. At the end of the session, the instructors helped to assemble two complete robots from the parts the students had built and demonstrated the operation of one functional robot.

Session 4: Manual control of robot
A functional robot was provided to each group at the beginning of session 4, i.e., four robots in total. The worksheet also contained a few questions (see chapter 8) to motivate the content to the students and to enable some basic assessment of whether the students gain understanding about the concept of density and what robots are useful for. Using the manual controls on the brick and push sensors, the students made a number of experiments: All groups could successfully operate the robot. Mixing of colors worked for all groups and everybody observed that one could not stack a denser fluid on top of a less dense one. Students had different success of whether to put the lower density on top, or push the higher density under – but all got positive results eventually. All students correctly predicted the final order in which fluids of different densities should be layered, and all students were able to do the layering by hand and by hand-controlling the robot.

Analyzing their work sheets including questions we found the following (not all students filled out all the questions): (1) We tested for potential learning gains, for example, whether the density-layering experiment increased conceptual understanding of why objects float and sink. Asking whether a person would float easier in a lake or an Ocean, 4/7 had correct answer in both pre and post-test, 2/7 went from no or wrong answer in the pre-test to the correct answer in the post test, 1/7 students had both pre and post-test wrong. 6/7 also mention salt as the reason for floating. Given the small sample size we cannot draw a generalizable conclusion, but the trend suggests that this activity could have a positive effect on delivering the density concept. We tested for understanding regarding the utility of pipetting as well as the advantages and disadvantages of robots: (2a) We asked for why pipetting is important for scientists, to which students responded "because it is", "being more precise," "not spilling," and "liquids don't need to be touched." (2b) We asked for the advantages of robots, to which students responded: "stronger," "more controlled," "help us," "can move without stopping," "can handle toxic stuff," and "can mix colors." (2c) We asked what the disadvantages of robots are: "slow," "can have glitches," "can break," "can't talk," "can get a virus," [presumable the student

meant a computer virus], and “you have to fix it.” Hence overall, the students made reasonable associations about why scientists move fluids, what robots are good for, and what disadvantages of robots are.

Session 5: Programming of robot
Each group had a computer with the *Lego Mindstorms EM3 Home Edition* installed on it to program their robots. From the eight students, one was absent during session 5 and one gave no consent to use her data. 3/6 have programmed Lego Mindstorms before; 2/6 did not, and 1/6 did not provide an answer. From their programming results, worksheets and our observations we found that all 6/6 students understood speed changes, 4/6 understood direction changes, 6/6 were able to move all 3 motors in a controlled fashion, 4/6 made a dilution series, and 2/6 made double dilutions with different step sizes series similar to figure 2H in the main paper. All groups implemented some sounds (e.g. to indicate the end of a program), which they had not been asked to do, but which one group started doing and then everyone followed suit with general amusement. One student pair lost interest in finishing these activities and starting doing unrelated things for the second half of this session.

After all five sessions we asked students what they had learned, leading to the following responses: ”That programming is fun.”; ” How to build, program, and use the robot.“; “I learned to build a robot.”; “Robots are cool.” ; “That robots can help us with every day things.“ We asked them what they like most from all 5 classes? “The layers.“; “”Everything!“; “Color mixing.“; “The robot“; “learning stuff.“ It is interesting to note that for learning they all refer to programming and robotics – but no answer related to liquid handling, while for liking two answers include refer to the actual experiments (layers / color) – and robotics are mentioned as well. Again the sample size is too small, but these answers suggest the trend that students perceived the learning primarily in the robotics / programming domain, while all components (the science experiments, colors, and robots) constitute motivating factors.

We also asked for how difficult the individual activities were on a 1-5 Likert scale (very easy, easy, medium, hard, very hard): programming (2.8 ± 1.5) (always mean ± stdev), building the robots (2.5 ± 0.8), density layering (2.5 ± 1.4), dilution series (1.8 ± 0.4), and color mixing (1.3 ± 0.5). Hence the building and programming perceived as slightly more challenging than the science activities, but overall the answers indicate that the level of challenge was well matched to the particular student group.

The overall rating of the course on a scale of 1-5 (very bad - brilliant) was 4.2 ± 1.0.

Conclusion and discussion on all five sessions:
Overall, these activities worked with 5th graders and the students were generally very engaged. It should be noted that these students do not represent the average 5th grader given that a significant number of them had already previous experience with Lego Mindstorms, furthermore they came from more affluent house-holds and schools.

The robots can be built by students but some guidance by instructors when students were occasionally stuck was needed. Had we let each group build their own robot in full, this would have likely required another one or even two sessions. And while the students clearly enjoyed building one or two modules, we expect that just building more parts by following instructions for multiple sessions in a row might not have appealed to all the students. Interspersing building with experiments while making the robot more complex over time (as we did by having students build the pipetting module in session 1 first, and then devoting session 2 to using it before going on to further building in session 3) certainly helped.

The progression regarding the mode of pipetting (Pasteur pipette, lab pipette, robot manual, programmed robot) and thereby reiterating on the same concept seemed to have worked very well. Being able to operate the robot manually (compared to programming it) leads to more immediate feedback for the student

The progression over five sessions covering different aspects of robotics, programming, and experimentation seemed to have worked well, and the provided worksheets (see section S7) should provide a good starting point for other instructors.

6.3. Third use case: Middle school students

The third use case explored how a single teacher could use these robots and STEM experiments with middle-school student (12 students; 11-13 years old; both genders; three students dropped out for the last four sessions due to time conflict with another school activity, they missed the final tests, hence much of the analysis focuses on the remaining nine; 8/9 had previous experience with Lego Mindstorms). The teacher was the same who had participated with the material development over the previous summer (and is also co-author on the paper). This middle school teacher regularly leads afterschool robotics classes in her middle school. She then adapted the content for 16 afterschool sessions (~30 h total). Each session lasted between 1 and 2.5 hours. The participating students were regulars of her robotics class. Students worked in groups of two or three. The students worked again with the 1D robot design (Fig.1A main paper). Compared to the deployment in elementary school, there was only a single instructor for a larger student number, the students worked under less supervision, all pairs built their own robot, they had more time for self-motivated side projects including individual changes to the robot design and experimenting, the teacher inserted several lectures about liquids, densities, and dilution factors. The teacher also designed and ran a post-test at the end of the course to assess aspects of student perceptions regarding liquid handing experiments, robotics, and the overall course. The teacher then also self-assessed the course success in relation to more established robotics activities, e.g., students building and programming a car using Lego Mindstorms.

The teacher laid out the following 16 sessions:

1. Unpack and familiarize with educational EV3 kit
2. Start building 1D robot
3. Finish building 1D robot

4. Start testing the 1D robot
5. Lecture on density and how it affects ocean currents, and buoyancy
6. Hand pipetting to explore the sequence of the liquids; automate when ready
7. Programming the pipette
8. Pipette fine tuned
9. Individual programs tested for optimization
10. One last testing session for reliability
11. Start overview on titrations
12. Dilution factor and volume of cuvettes
13. Dilution factor decided
14. Fine tune programs, discuss design
15. Post Engineering test; discuss programming
16. Clean-up and post-quiz

The course overall ran well, and all students were successful in executing these activities.

The post quiz assessed students' opinions regarding various aspects:

The challenge of the activities was reported as having medium difficulty (2.1± 0.9 – 3.3 ± 0.7) on a scale of 1-5 (very easy – very hard). The difficulty of the individual categories were rated as: Building the robot (2.1±0.9); Color mixing (3.1±0.8); Color dilution series (2.7±0.9); Density layers (3.3±0.7); Programming (2.9±0.8).

Do you feel more competent building with LEGO™ after this course? 7/9 answered yes.
Do you feel better able to program with LEGO™ after this course? 8/9 answered yes.

Did you learn something new during this course? 9/9 stated "yes" If so, what: 7/9 answers were related to wet-work. (All answers: "I learned a lot about teamwork, communicating with others, and about how scientists use pipetting"; "I learned about salt densities"; "How to build pipettes"; "I learned about density and that density=Mass/volume"; "Building"; "I learned what a serial dilution was"; "I learned what serial dilution is"; "I learned about serial dilution"; "I learned how robots can be helpful in daily life")

What have you learned about robotics? Student gave a variety of answers, 5/9 can be interpreted that the course widened the student's view of robots can be used for. (All answers: "Robotics is a way for student to learn and develop"; "I learned that robotics, even EV3's can be used in different ways"; "It is a lot more impressive than moving robots"; "Robotics is about finding efficient, more accurate ways to accomplish a goal"; "LEGOs can be used for good things"; "I learned how to program a pipette"; "I learned about programming & Building a non-LEGO object like a pipette"; "I learned that robotics can be applied in the real world in disciplines which don't focus on engineering"; "Robotics are a necessary skill")

What have you learned about liquid solutions? (All answers: "I found out which of the solutions is denser"; "liquids with higher salinity fall"; "nothing"; "When pipetted

carefully into a container, liquid solutions can settle into separate, discrete layers depending on their individual densities"; "It's not easy"; "Serial dilution can be helpful in real life"; "They (solutions) can have different salt densities"; "I learned how to apply the D=M/V to density"; "A solution requires care to make")

What have you learned about liquid handling? (All answers: "When handling liquids it is important to make precise measurements or your results will not be accurate"; "To be very precise"; "With a liquid containing salt, shaking the liquid in the container will help dissolve excess salt crystals"; "I learned that some solutions have different weight"; "I learned how to create a serial dilution"; "I learned how careful and precise you have to be"; "Pipettes were used to measure exact amounts of liquid. Before, I thought that pipettes were the things used in kitchens"; "You need to be careful not to add too much of a mixture")

When asked *what would happen if a sample of red water with a high salt content and a sample of green water with no salt, and then put one over the other* (both options), 9/9 provided the correct outcome. When asked to give an explanation, 6/9 correctly referred to the different densities, 2/9 referred to difference in the amount of salt.

As a transfer questions, the students were then asked where it is easiest to swim ("float"): In a lake with fresh water; lake with salt water; or lake with muddy water, only 5/9 gave the correct answer (salt water lake due to higher density). Some of the wrong answers also hint at other misconceptions, e.g., "Mud is dense, and the denser the liquid the more you can float in it" (likely confused with optical density) and "Salt water would have more dense water, and harder to swim through" (likely confused "floating" with the effort to actively swim through a dense medium). Hence although some students correctly answered this question (and some students also self-reported learning about density, and all students correctly recapitulated the density layering experiment), this concept would require more care in scaffolding to avoid various misconceptions. We also note that the concept of density is only taught in 6th grade, i.e., some of the students had not yet had it in class (students were a mix of 6th, 7th, and 8th graders).

Students were asked to name three advantages and disadvantages of robots: We categorized answers if possible (total number of mentions in brackets). Advantages: Being very precise and accurate and not making any mistakes (10); Makes human work live easier by saving time and replacing menial tasks (7); More efficient and faster than humans (4); Other single mentions: "They can go places we can't"; "Robots can serve as guardians of teachers"; "Robots can copy different actions"; "We can fix anything made by robots using interchangeable parts"; "They can save lives"; "Can help people with sicknesses." Disadvantages: Cost to build and maintain (7); Lots of effort to build and maintain (8); Malfunctions, e.g., software bugs (6); Other single mentions: "Replace people's jobs"; "They can be used to do bad things"; "Certain aspects can make life harder"; "Isn't creative"; "has no feelings - unreachable for human"; "Robots are known to backfire.") It is interesting to note that there was only a single reference to the actual liquid handling (Quote for advantage "Less work to pipette solution"). To what extent

these opinions were formed during this particular course, or already existed before, is not possible to say.

Do you think girls or boys like this course better? 9/9 stated that course would appeal equally to both. (All answers: “We all enjoyed this course and we were happy to work with each other”; ”I think it depends on a specific person's interest”; “Gender should not matter. it just depends on the type of person you are, scientist or not”; “Our group was all girls while the other 3 groups were all boys but we all enjoyed the course equally”; “So both can help each other”; “Your gender does not impede you from doing this course in any way”; “I don't think gender matters with something like this”; “It doesn't matter what gender you are to like this course”; “Anyone can have fun with LEGO Mindstorms”)

What did you like most from all sessions? (All answers: “I like to work as a team and program together”; “I like the serial dilution the most”; “The moments when we all cooperated in peace and happiness”; “In most of the sessions, there was a lot of new things to be learned - each session was a learning experience”; “Programming”; “I liked programming our first experiment”; “I like the density layers sessions the best”; “I liked building and programming the robot”; “I enjoyed experimenting with liquids and seeing what liquids do”)

This course was rated 4.2 ± 0.4 on a scale of 1-5 (strongly unfavorable to strongly favorable). When students were asked to rank five main activities based on how much you liked them (Most favorite=1, Least favorite =5), the rank ordering was very different between students, leading to average ranks of: Building the robot (2.3 ± 1.4); Density layers (2.8 ± 1.6); Programming (3.0 ± 1.7); Color mixing (3.2 ± 1.2); Color dilution series (3.7 ± 1.1). (Every item was rated lowest by at least one student, and every item was also ranked highest by at least one student, except for the dilution series which was ranked 2nd by at least one student.)

Teacher’s assessment and reflections

We interviewed the teacher after the course to reflect on what worked well, what could be improved in the future, and how these activities compare to the more traditional robotics afterschool activities. We took notes and then summarize the following points:

How did the course work overall?
The teacher stated that the course overall went well, that the material was suitable, and that the students were engaged. In order to motivate the students of what to expect, they were shown initially the pictures of the final robot, the density layer experiment, and the dilution series experiment. Building from the Lego digital designer worked well and students could assemble everything independently. Given their previous experience with Lego Mindstorms, the main new “engineering challenge” for this student cohort was the programming of loops. Given that the students were a mix of 6th, 7th, and 8th graders, some had learned about the density concept already in school, and for others it was new; giving additional lectures on this concept helped. Students did not seem to be too interested in working with the hand-held pipette or operating the robot via pushbuttons,

instead they were much more interested in programming the robot and then letting the robot execute these experiments. Overall the students liked the science activities.

How do these activities and outcomes compare to other robotics projects you have done?
The teacher stated that the typical Lego NXT/EV3 project is the "car like motion robot," which is primarily about moving around a field and potentially picking up and transporting other physical objects. The liquid handling robots are unique in that they can do science experiments, and students were very intrigued by that, and also voiced that they had never seen something like that before and that it is interesting that one can do science with robots. The pipette has many versatile affordances, especially as a number of different experiments can be done. A "robot car" focuses more on physics and engineering, while a "liquid handling robot" branches more into science. This new robot also teaches students more of the concepts of "metered" liquid handling, e.g., how to get a serial dilution to work, how fast to dispense etc. The classic programming and engineering skill are certainly tested and expanded with the liquid handling robot as well. (Note also the two student quotes from the post quiz: "I learned that robotics, even EV3's can be used in different ways"; "It is a lot more impressive than moving robots.")

What were the main challenges?
The teacher indicated that overall the activities worked very smoothly. Sometimes students had challenges with identifying the lengths of some axles (which is a limitation of the design program, and we consequently introduced that information explicitly into the building plans – see Table S1); having additional photographs of the setup as backup also helped the students. Students within groups typically split up, each building individual modules. Students made their own modifications, for example they took out the gearbox (Fig. S10), and instead used these pieces to build a direct indicator that counts the degrees of rotation of the motor driving the card, which then made it easier to determine how many degrees the motor needed to advance for certain tasks (e.g., traversing the distance between cuvettes) when actively programming more complex experiments. For some students the ruler was slightly curved, and the tape was too sticky, so they used extra Lego pieces to put the cuvettes onto a different surface, and also used standard double sided tape that was not as strong. Students also used some additional pieces to make some parts of the robot more bit sturdy (but all these pieces came from the EV3 expansion pack). It would also be nice to incorporate more sensors onto the 1D robot, as the color sensor was not very reliable in detecting color concentrations.

What is the right target group for these activities?
The teacher indicated that 7th-9th grade might work best; the NGSS introduces the density concept around the middle of 6th grade, and then again in 8th grade with the topic of characteristics of liquids. Ideally, students should have built a mobile car robot at some earlier time before working with the liquid handling robot. The girls in the group felt equally confident as the boys, and the boys also really wanted to do these activities as it was different and as it was challenging their programming skills. Overall, this was a self-selected group of students as all these activities were voluntary. These liquid handling robots and science activities expand students' existing concepts of what robots are about

and this could potentially also draw in more girls, but it is not clear at this point whether that would truly happen.

Conclusion middle school deployment:
We conclude that the deployment went well, in particular it can serve as complement or enhancement to the general middle school curriculum that robotics and science teachers can deploy, likely making additional modifications towards specific needs. These liquid handling robots can extend the students' perception of what robots can be used for.

6.4. Conclusions all user testing

Reflecting on all three deployment scenarios, we conclude that these liquid handling robots have versatile affordances and are suitable for a range of target audiences, instructional goals, and durations of these activities. In particular, the cross-over between robotics and science experiments was considered unique and novel compared to existing activities, such as building the "robot car."

The deployments went well, in particular experienced robotics and science teacher could deploy these setups in middle school, likely making additional modifications towards specific needs. We note that the middle and elementary school deployment had a very different pace. Students self-reported learning – both related to science / liquid handling experiments as well as robotics. All participant groups were certainly biased as a significant portion had been exposed to Lego Mindstorms before, and/or came from above average affluent schools and households.

The most suitable target audiences are potentially middle school (6th- 8th grade), but very focused activities with younger students are possible, as well as much more extended, open ended projects with high-school students. Depending on scaffolding and how much the teacher already prepares, a stand-alone activity only lasting one or two hours is possible; but overall we see the main potential to utilize these activities on a longer time scale for project based learning while integrating science and robotics themes.

We were gratified to see that these activities seem to appeal to both genders as increasing gender diversity in the STEM areas is important. Our observations with the girls in the elementary school suggests that the usage of different colors as well as liquids seems appealing - as also indicated by the fact that students often repeated experiments on their own initiative using different / "their favorite" colors. Similarly, the students in the middle school voiced the opinion that these activities would appeal equally to girls and boys. Whether this combination of liquid handling robots and STEM experiments is particularly suited to bridge gender barriers (potentially in both directions) will require further studies.

What needs to follow now is usage, deployment, and adaptation by other teachers and students, accompanied by educational researchers to establish best practices, also focusing on a more diverse demographics. The material (robots as well as science experiments) has a sufficiently low entry barrier to support that.

7. Example worksheets as used with Girl Scouts (elementary school)

Day1: Overview, manual mixing, building of pipetting head module

Worksheet Day 1 – Overview Lego Robots

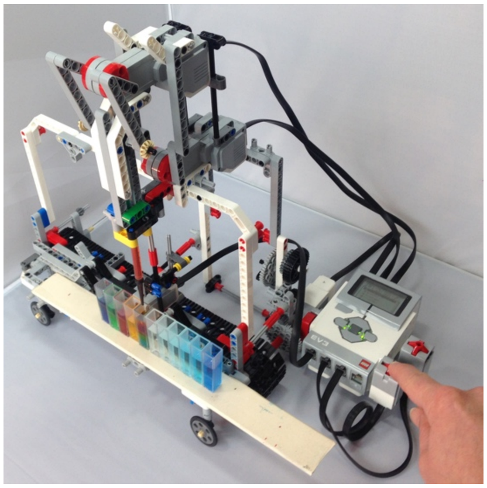

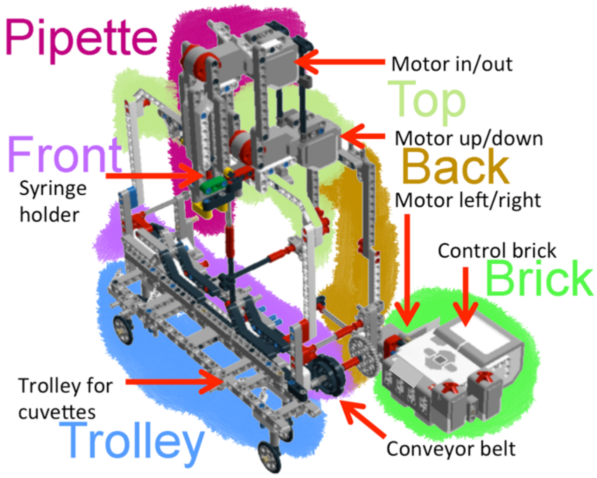

1. **Build pipette head.**
   According CAD file.

2. **Add syringe as shown on separate handout.**

3. **Build Brick with two push buttons to control the pipette.**

4. **Upload code with help of instructor.**

5. **Connect your pipette head with the Brick.**

6. **Test to move the motor.**

Day 2: Use of robot's pipette head module only

Worksheet Day 2 – Manual pipetting + Building

1. **Finish pipette head.**
   According to CAD file.

2. **Build the control brick**
   Use computer file 6Brick.lxf to build

3. **Connect motor to port C. Connect push buttons to port 2 and 3.**

4. **Upload code with help of instructor.**

5. **Test to move the motor.**
   Question: What was the hardest part during the building?

   Answer:
   ____

6. **Pipette liquids.**
   Note: 1 button is fast, 1 is slow. Why do you think is this important?

   Answer:
   ____

7. **Mix two colors into one cuvette.**
   What colors did you pick, and what did you observe?

   Answer:
   ____

8. **Experiment A: Dilution series**
   - Fill one cuvette (the small plastic container) with colored water 75% full.
   - Fill tap water into 5 more cuvettes 75% full.
   - Dip pipette tip into the colored water.
   - Fill syringe by pressing touch button.
   - Move tip to second cuvette and empty syringe by pressing the button.
   - Fill/empty twice to mix the solution.
   - Repeat in series to obtain something similar to the picture below.

   Dilution series result

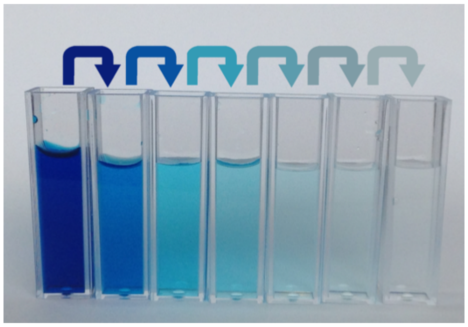

How many dilutions have you made?

Answer:

What is the dilution factor from one cuvette to the next cuvette?

Answer:

What is the dilution factor from the first to the last cuvette?

Answer:

---

9. **Experiment B: Density stacks**
   - Obtain 2 colored liquid form the instructor.
   - Try to layer the 2 solutions so they stay separated.
   - Tip: Try different order of pipetting.
   - Tip: Try different speeds of ejection.
   - Tip: Try bottom-up vs. top-down.

   Density layer results

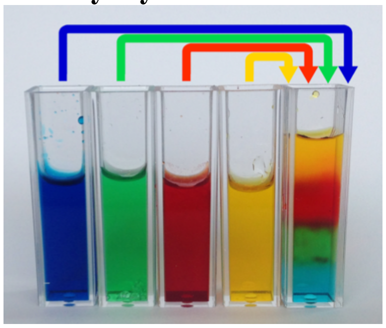

What was your strategy so the liquids mixed the least?

Answer:

Why do the two fluid mix sometimes and sometimes not?

Answer:

---

10. Check with instructor to build rest of robot

Day 3: Building complete robot

Worksheet Day 3 – Lego pipetting robot

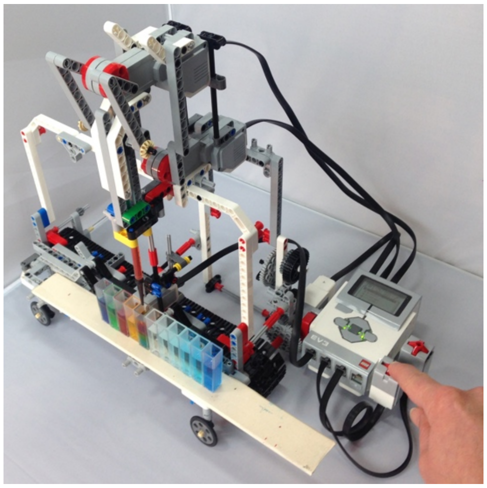

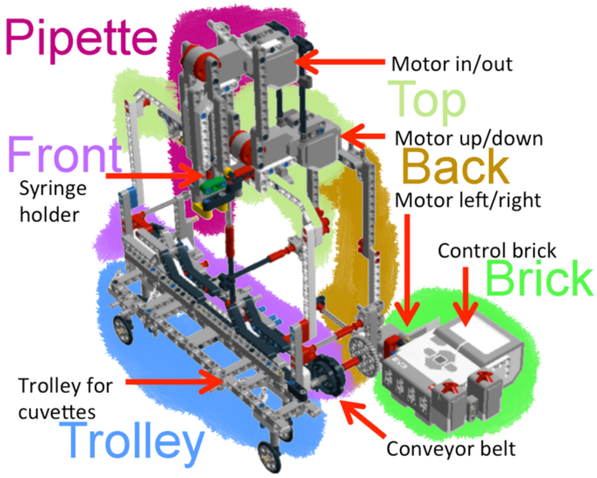

Open a *.lxf CAD file with the Lego Digital Designer software and switch to building mode (F7) to build one or two robot modules by following the step-by-step instructions. The instructor will help assembling all parts to a full robot.

Day 4: Manual control of full robot

Lego pipetting robot – Worksheet Day 4

A) Questions

- What floats best in water: □ A gold bar, ☐ block of wood, or ☐ a human swimmer?
- What floats worst?
- Why?

  Answer:

  ____

- Where do you float easier? In ☐ a lake or ☐ the Pacific?

- Why?

  Answer:

  ____

- Do you have a Lego robot at home? ☐YES ☐NO

- Have you ever taken a robotics class before? ☐YES ☐NO

B) Check if your robot is working properly

- Can you run the program called “Manual”? ☐YES ☐NO

- Can you move the trolley left/right? ☐YES ☐NO

- Can you lift/lower the pipette head? ☐YES ☐NO

- Can you fill/empty the syringe? ☐YES ☐NO

C) Manual operation – color mixing:

- Place 3 cuvettes on the trolley. Make sure they are aligned with the pipette tip: They should be just below the pipette tip.

- Pick two colors and fill them into two cuvettes. About 80% full.

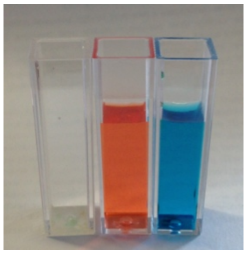

- What colors did you pick?

  Answer:
  ____

- Use the program called “Manual” to mix the two liquids in the third cuvette.

- What color resulted after you mixed them together?

  Answer:
  ____

D) Manual density layers:

- The instructors will give you 4 solutions with different salt content. The difference in salt content influences the density of the liquids.

- Which of the solutions will be the densest and therefore sink to the bottom?
  ☐ high salt content, ☐ medium salt content, or ☐ low/no salt

- Do a 1st experiment by using a plastic pipette and try to layer blue and green.
  1: blue vs. green
- Try to put in blue first. Then put green under it. Does it work? ☐Yes ☐No
- Try to put in blue first and the put green on top. Does it work? ☐Yes ☐No
- Try to put in green first. Then put blue under it. Does it work? ☐Yes ☐No
- Try to put in green first and the put blue on top. Does it work? ☐Yes ☐No

- Try to fill in the second solution on the top or to the bottom. You can eject the second solution to the top of the first solution or to the bottom.

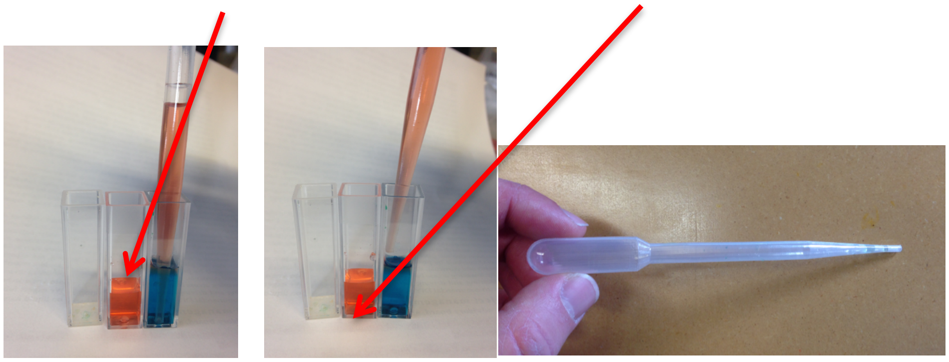

- What works better? Second on top or bottom?

  Answer:
  ____

- Why does one solution say on top of the other one?

  Answer:
  ____

- What is the stable order? Which color floats best?

  Top: _____________

  Bottom: _____________

- Call instructor to check if you layers look good.

Now, let’s use the robot to test the other colors

- Align the 3 motors as shown in the picture below:
  - 1) Empty syringe
  - 2) Pipette all the way up
  - 3) Pipette tip above colored cuvette

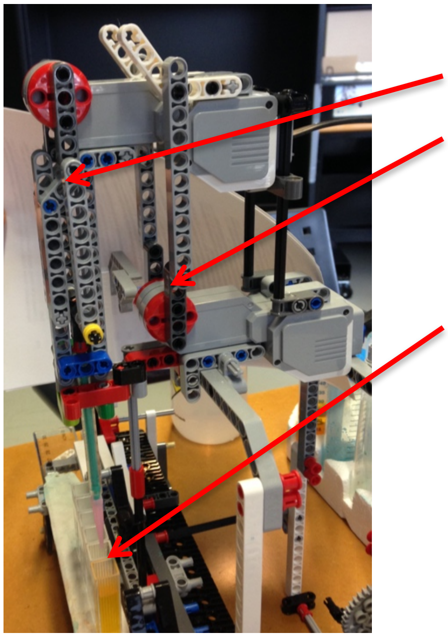

1) Empty syringe. Plunger all the way in.

2) Pipette all the way up.

3) Pipette tip is just above the right syringe.

- Use the program “Manual” to manually transfer two solutions into a third well.
- To eject the liquid, you should dip in the pipette tip completely into the cuvette.

- 2: blue vs. red

  Top: ______________
  Bottom: ______________

- 3: green vs. red

  Top: ______________
  Bottom: ______________

- 4: green vs. yellow

  Top: ______________
  Bottom: ______________

- After tests 1-4: can you tell in what order the colors form stable layers if you add all four into one cuvette?

  Top: _____________

  Second: _____________

  Third: _____________

  Bottom: _____________

- Try to layer all four liquids in one cuvette **by hand**.
  In what order should you do this?

  Answer: ____

- **Were you able to layer all four solutions in one single cuvette by hand?**

  Answer: ____

- Call instructor to check if you layers look good.

- Now try to layer all four liquids in one cuvette by using the **robot**.

- **Were you able to layer all four solutions in one single cuvette with the robot?**

  Answer: ____

- Call instructor to check if you layers look good.

E) Now let's use the robot to do all the work! Automated density layers:

- Call the instructors to help you upload the code for the automated layers.
- Prepare 5 cuvettes according the instructor's instruction.
- Run the program "Layers".
- **Describe what happened:**

  Answer:

  ____

- **Were there any problems?**

  Answer:

  ____

- **Were you able to make the automated density layers?**

  Answer:

  ____

- Call Instructor to check if you layers look good.

G) Final questions:

11. **Density**

    Where is it easier to float: in a lake, the pacific, or the Dead Sea?

    ☐Lake ☐Pacific ☐Dead Sea

    Explain your pick.

    Answer:

    ____

12. **Why do you think pipetting is important for scientists?**

    Answer:

    ____

13. **Why are robots important to us?**

Answer:

14. Name three advantages of robot?

1.

2.

3.

15. Name three disadvantages of robot?

1.

2.

3.

Day 4 – Optional worksheet

H) Automated dilution series:

- Check with instructor before continuing.
- Clear out all cuvettes by using a plastic pipette.
- Place a total of 6 empty cuvettes on the ruler. Make sure they stand close together and are aligned with the pipette tip. See picture below.
- Fill the right cuvette with your favorite color to about this height (80% full)
- Fill the other 5 cuvettes with water to about this height (70% full)

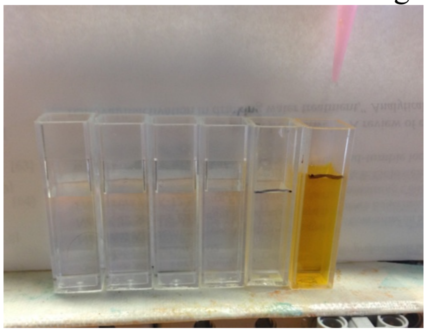

- Call the instructors. They will check your setup and help you to upload the code.
- Was your setup correct? ☐YES ☐NO
- The instructors will now help you upload the code for the automated dilution series.
- Run the program called "Dilution series".
- **Describe what happened:**

  Answer: ____

- **Were there any problems?**

  Answer: ____

- **Were you able to make the automated dilution series?**

  Answer: ____

- Let's do another dilution series with another color!
- Use a plastic hand pipette to empty all cuvettes.
- Prepare as above (color in the far right cuvette and water in the others; align properly).
- Do you want to do it by ☐ hand pipette, ☐ manually, or ☐ automated? Check one.

Day 5: Programming

Lego pipetting robot – Worksheet Day 5

Your name:

1. **Pre-quiz**
   Have you programmed Lego Mindstorms before? ☐YES ☐NO

2. **General program:**
   - We use *Lego Mindstorms EV3 Home Edition* software.
   - Open the program.
   - Click File > New Project
   - You will see the *Start* block on an empty page

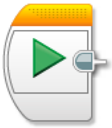

3. **Learn to move one motor:**
   - Select a small motor (green tab, left icon)
   - Click it and drag it next to the *Start* block.

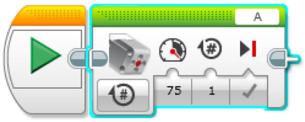

   - You can now change the port, speed, and direction.
   - Follow the cable from the lowest motor (the one that drives the belt) and see to what port it is plugged in. It should be A.
   - Make sure you can see a small A in the top-right corner.
   - Click on the 75 to set the power (=speed)
   - Set it to 10.
   - Click on the left round arrow and select "On for degrees".
   - Click on 360 to set the distance to move.
   - Set it to 50.

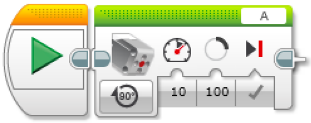

This is how it should look like:

4. **Connect the robot to the computer**
    - Use a USB cable
    - Switch on the Lego Brick by pressing the middle grey button.
    - On the computer you should see this in the bottom left corner:

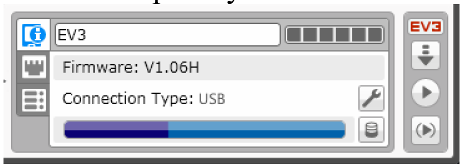

    - Click on the Play button.
    - Describe what happened:

____

5. **Explore the functions**
    - Change the speed to 20. Run the program again.

      What happened?

      ____

    - Change the speed to -10 and change the degree to 40. Run again.

      What happened?

      ____

    - Try different combinations. Use speeds from -50 to +50.

      What did you observe?

      ____

    - What happens if you take very big (e.g. 1000)?

      What happened?

      ____

6. **Let's program the pipette head lift**
    - Click the green part of the motor and drag it away from the Start Block
    - Select a new big motor from the selection at the bottom:

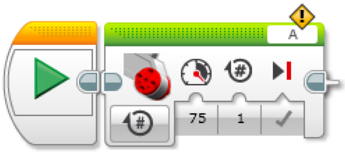

    - Change port to B.
    - Change operation mode from # (rotations) to degrees (90°).
    - Change speed to 10.

- Change degrees to 180.

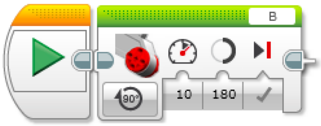

It should look like this now:

- Run the program by clicking on Play-Button in the bottom right corner.

- What happened?

____

7. Let's program the syringe motor

- Disconnect the previous block from the Start block
- Add a new motor block to the Start block

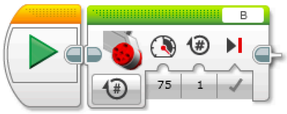

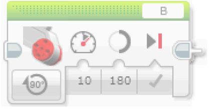

- Change port to C.
- Change operation mode to degrees.
- Change speed to 10.
- Change degrees to 180.

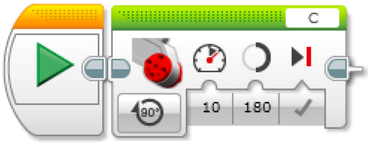

- Run it.
- What happened?

____

8. Combine all three motors in one program

- Make one liquid transfer with the following program:

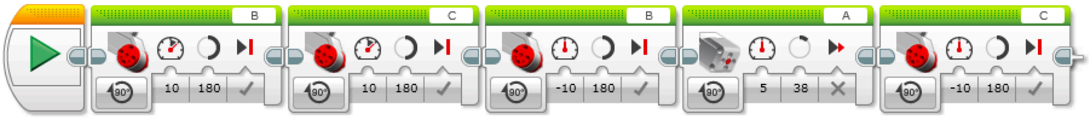

- You can use the blocks you made before or generate new ones.
- Make sure all the values match the picture above.

- Align all the motors to position zero (Plunger inside cuvette, head up)
- Run it again.

9. Now let's use liquids!

- Place two cuvettes onto the ruler next to each other.
- Fill the right cuvette with a colored liquid.
- Align all the motors to position zero (Plunger inside cuvette, head up, tip over most right cuvette.
- Run your program again.

- What happened?

  ____

10. Dilution series

- Now let's make an automated dilution series.
- Remove all cuvettes.
- Place 5 cuvettes next to each other on the ruler.
- Fill the right cuvette with a colored liquid.
- Fill the other 4 cuvettes 75% with water.
- Align all the motors to position zero (Plunger inside cuvette, head up, tip over most right cuvette.

- We want the same program as above to run 4 times.
- You could just start it 4 time in a row, or…
- Let's insert this loop!

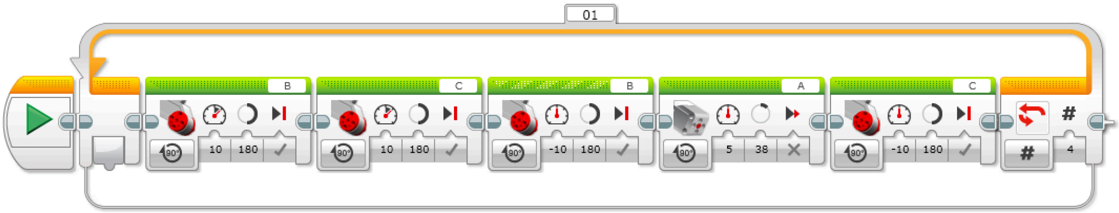

- Run the loop.

- What happened?

  ____

11. Let's skip every second cuvette.

- Now we want to dilute only every second cuvette.
- To do so, we need to move the trolley (motor A) two times.

- Make inner loop like this:

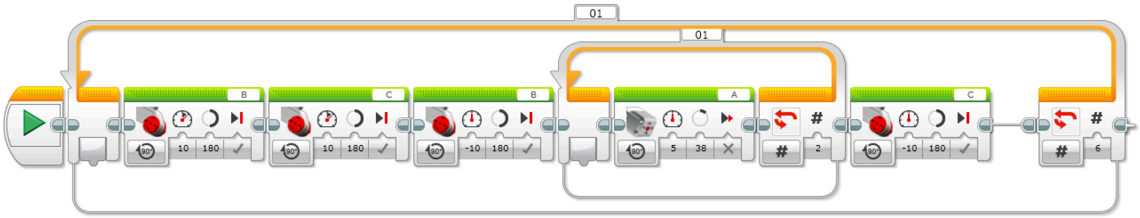

- Place 12 empty cuvettes.
- Add water (about 20% full only. See image below)
- Choose a color and place it to the far right.

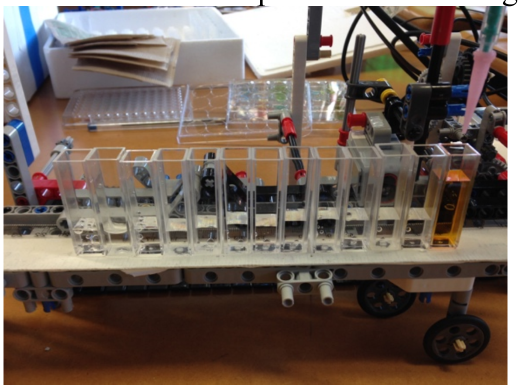

- Align all motors. Start program.

- What happened?

____

- Result: 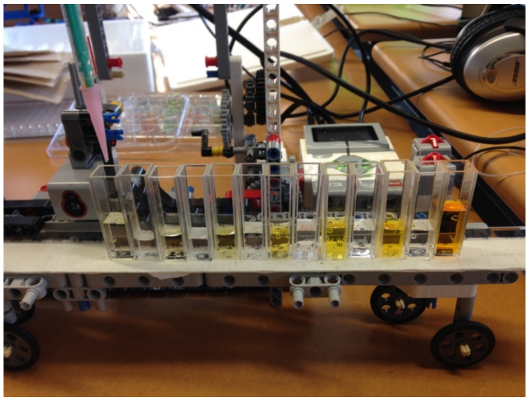

12. Let's skip two cuvettes with another color.

- Change the two numbers for the loops according to this image:

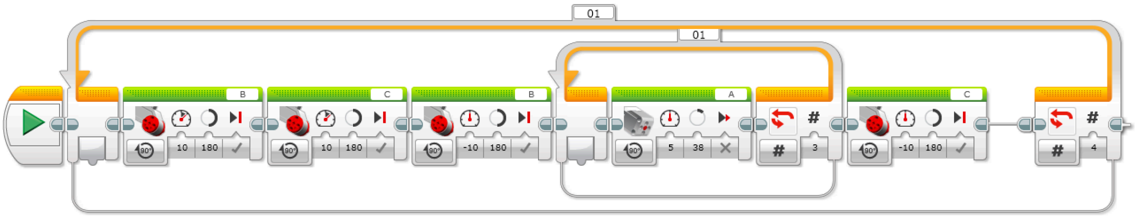

- Exchange the liquid in the far right cuvette (containing the pure color) with another color.
- Align again and run the program.

- What happened?

____

- Start:

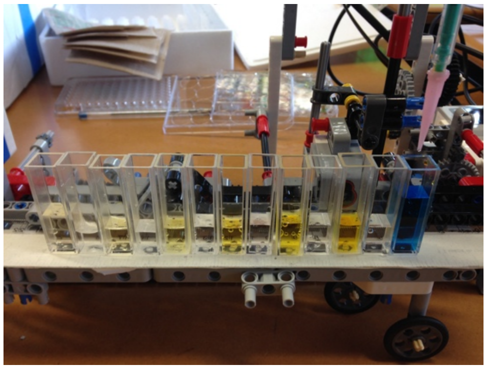

- Result:

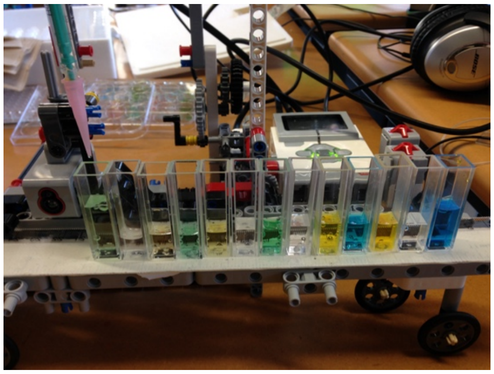

13. Let’s skip three cuvettes with a third color.

- Change the two numbers for the loops according to this image:

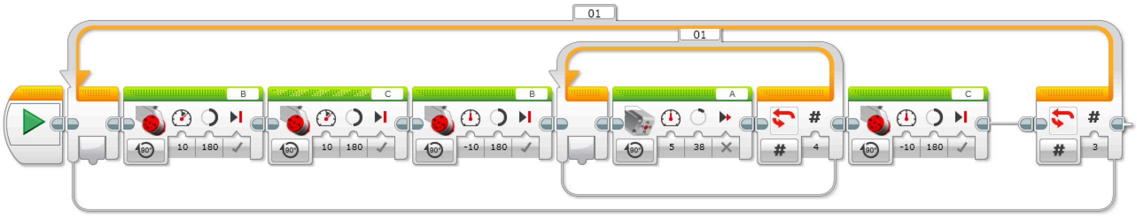

- Exchange the liquid in the far right cuvette (containing the pure color) with another color.
- Align again and run the program.

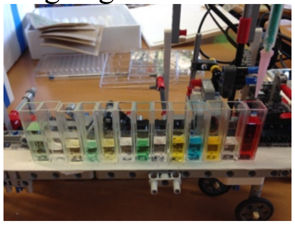

- What happened?

____

- Result:

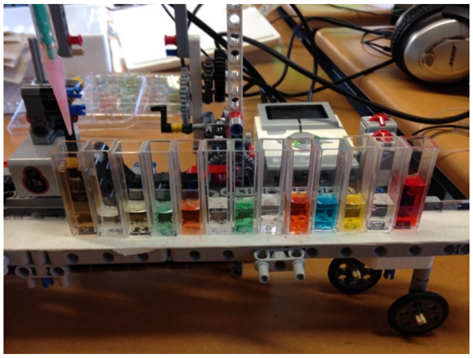

- **Questions:**
- What cuvette numbers remained with no color?

  Answer:

  ____

- What is a computer program?

  Answer: ____

- Which block can be used to make a loop?

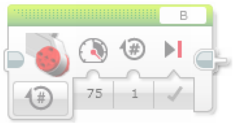 ☐ 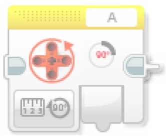 ☐ 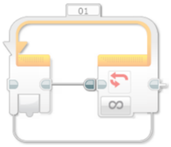 ☐ 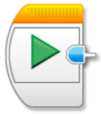 ☐

14. Post-quiz overall

- What did you learn during the last 5 robotics classes?

Answer: ____

- What did you like most from all 5 classes?

Answer: ____

- How did you like overall these 5 robotic classes?

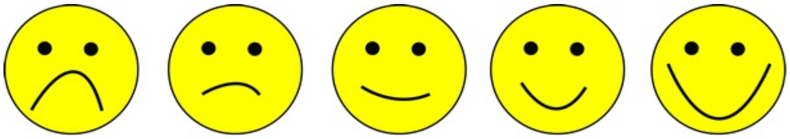

Very bad ☐ bad ☐ neutral ☐ good ☐ ☐ brilliant

- How difficult were these activieties?

Building the robot: ☐Very easy ☐Easy ☐ Medium ☐Hard ☐Very hard

Color mixing: ☐Very easy ☐ Easy ☐ Medium ☐Hard ☐Very hard

Color dilution series: ☐Very easy ☐ Easy ☐ Medium ☐Hard ☐Very hard

Density layers: ☐Very easy ☐ Easy ☐ Medium ☐Hard ☐Very hard

Programming: ☐Very easy ☐ Easy ☐ Medium ☐Hard ☐Very hard

Colors on paper: ☐Very easy ☐ Easy ☐ Medium ☐Hard ☐Very hard

- Rank the following activities how much you liked them. Best=1, Worst=6
  Best=1:
  _____ Building the robot
  _____ Color mixing
  _____ Color dilution series
  _____ Density layers
  _____ Programming
  _____ Colors on paper

Additional Worksheet – Calibration and Sensor of 1D robot

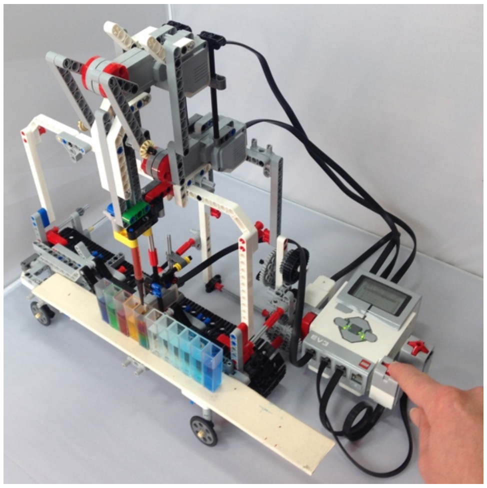

This worksheet explains how to align motors and use the color sensor for concentration readout. This fosters more quantitative approaches and measurement concepts.

1. **Dry-mode**
   Before you start with any liquids inside the syringe and cuvettes, you should make sure that your robots operates correctly in "dry-mode".

2. **Filling the syringe**
   To fill the syringe completely, you can always us a 180° turn from the empty position.

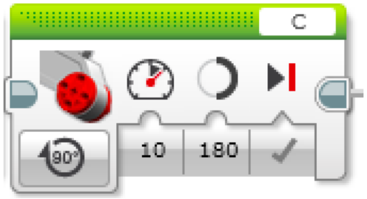

3. **Release liquid**
   To release liquid, you can turn any degree from 1°-180° to release the desired amount. Please note, that not every degree results in the same amount released! This is due to the theoretical issue that the motor rotates at a constant speed that is translated to a non-linear speed of the plunger (sinusoidal behavior) as well as practical issues such as trapped air inside the syringe which leads to a delayed release due to the air's compressibility. Also will different syringes and syringe tips have an influence on the amount of liquid released per degree turned. Therefore it is recommended to measure and calibrate the syringe routine to your needs. In general it is safer to release liquids slower than taking them up.

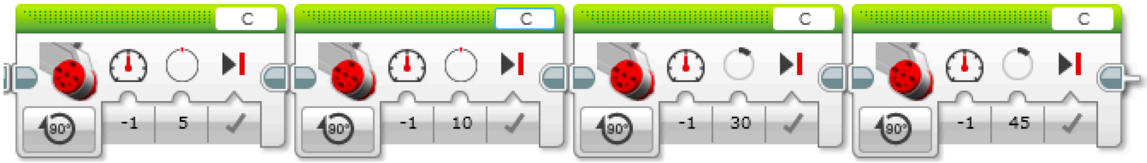

This sequence will release 4 different amounts of liquid and the syringe will be about half full afterwards.

4. **Lower and lift the pipette head**
   The sequence below will lower the pipette head by 110° and then rise it again to the top in the other direction. If you want to go all the way down, insert 180 instead of 110.

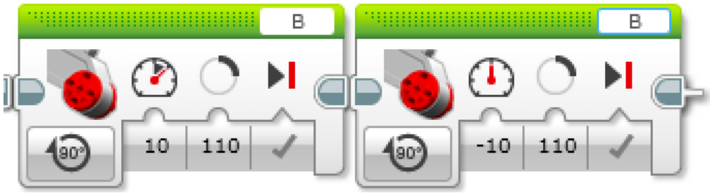

5. **Move the trolley**
   To find the right distance that the trolley has to move from one to the next cuvette, you can align the pipette tip over one cuvette and manually move to the next cuvette. The integrated sensor (bottom right corner in the Lego software) shows you how far you have travelled. This is the amount you can use to automatically travel from one cuvette to the next.

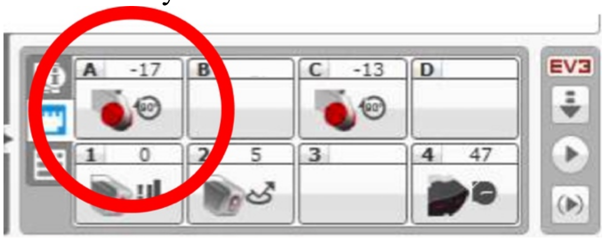

6. **Color sensor**
   To use the color sensor to read out concentrations as shown below you must place the sensor in the right place in the back of the cuvette. They must be aligned perfectly to make sure that only one cuvette is read. Also make sure to only readout full cuvettes, as much area is needed for a good readout. Experiment with changing the distance of the sensor to the cuvette, the lighting conditions, steady lighting condition, and try placing a steady background (such as some cardboard) on the opposite of the cuvette. Let the robot acquire the concentration read outs multiple time and then take averages.

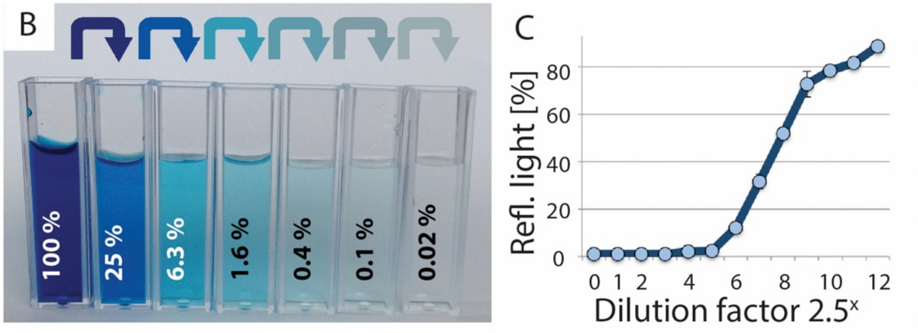

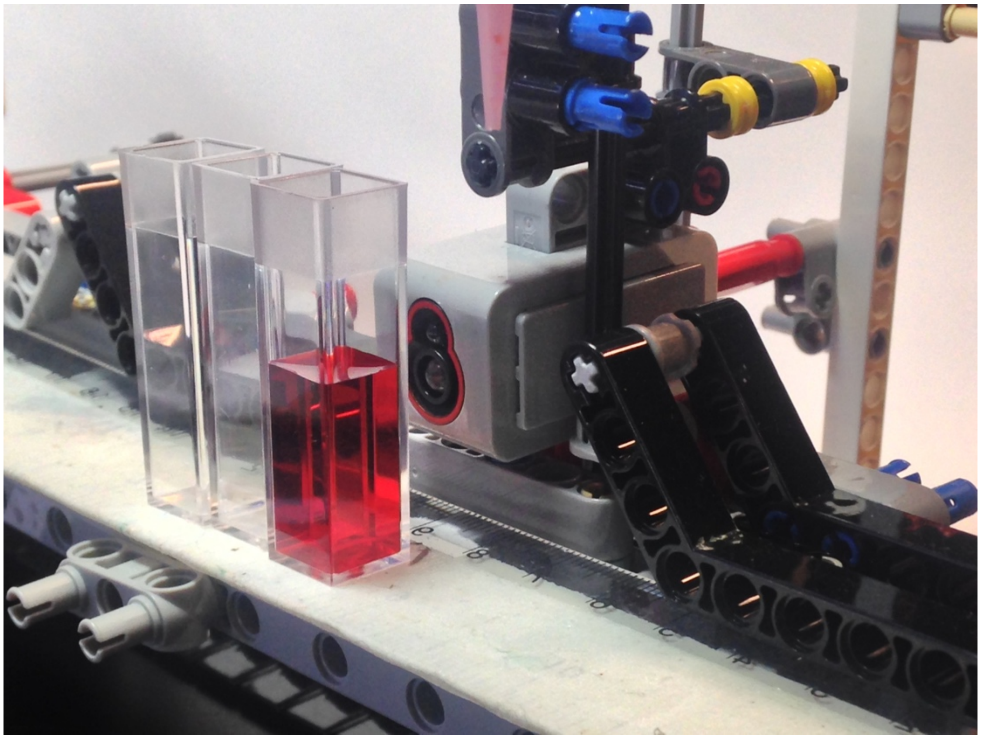

Sensor placement as shown typically works, but it is recommended to read out only full cuvettes. The ones shown here are only about three quarter full.
